# Supplementary material for: Factors associated with hypertension in Pakistan: A systematic review and meta-analysis
Source: PLoS One. 2021 Jan 29;16(1):e0246085. doi: 10.1371/journal.pone.0246085 (PMC7845984; doi:10.1371/journal.pone.0246085)
Supplement: S1 Fig — (DOCX) [file pone.0246085.s001.docx]

**S1 Fig : Funnel plots assessing publication bias in the results for age-groups**

**1_1 Age group: 30-39**

**
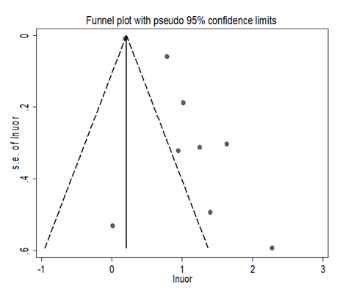
**

**1_2 Age group: 40-49**

**
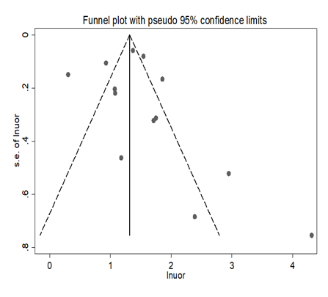
**

**1_3 Age group: 50-59**

**
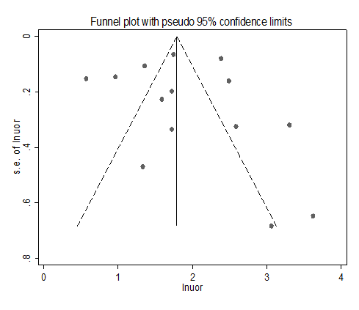
**

**1_4 Age group≥60**

**
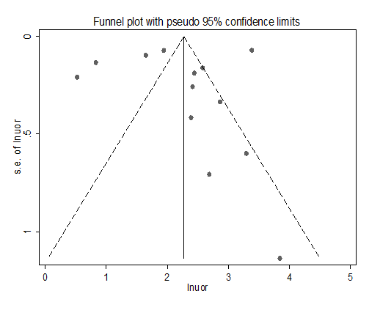
**
