# Supplementary figures and images for: Factors associated with hypertension in Pakistan: A systematic review and meta-analysis
Source: PLoS One. 2021 Jan 29;16(1):e0246085. doi: 10.1371/journal.pone.0246085 (PMC7845984; doi:10.1371/journal.pone.0246085)

**S2 Fig : Forest plot from the Meta-analysis (using Random Effect Model) of gender**

**
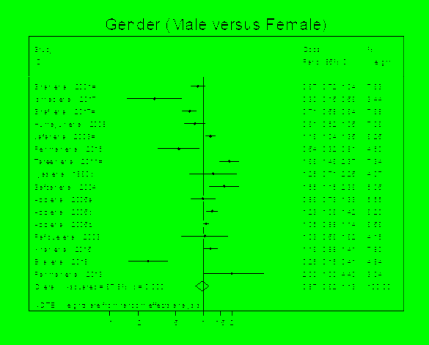
**

Supplement: S2 Fig — (DOCX) [file pone.0246085.s002.docx]

**S3 Fig : Funnel plots assessing publication bias in the results for gender**

**
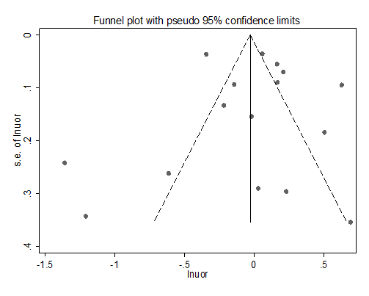
**

Supplement: S3 Fig — (DOCX) [file pone.0246085.s003.docx]

**S4 Fig : Forest plot from the Meta-analysis of marital status**

**
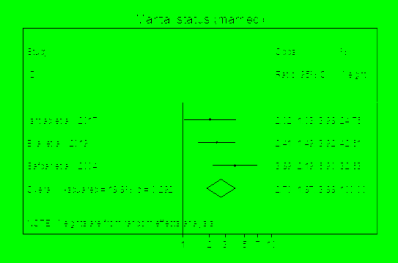
**

Supplement: S4 Fig — (DOCX) [file pone.0246085.s004.docx]

**S5 Fig: Funnel plots assessing publication bias in the results for marital status**

**
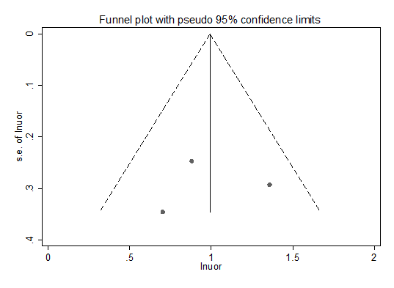
**

Supplement: S5 Fig — (DOCX) [file pone.0246085.s005.docx]

**S6 Fig : Forest plot from the Meta-analysis of the levels of education**

**
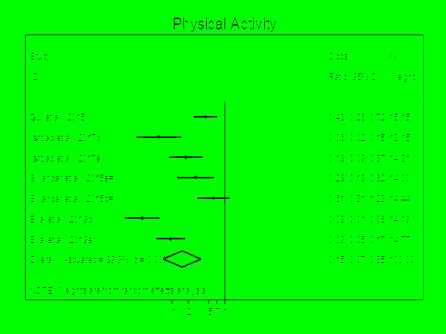
**

Supplement: S6 Fig — (DOCX) [file pone.0246085.s006.docx]

**S7 Fig : Funnel plots assessing publication bias in the results for the level of education**

**
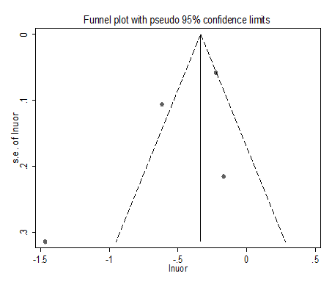
**

Supplement: S7 Fig — (DOCX) [file pone.0246085.s007.docx]

**S8 Fig: Forest plot from the Meta-analysis of income status**

**
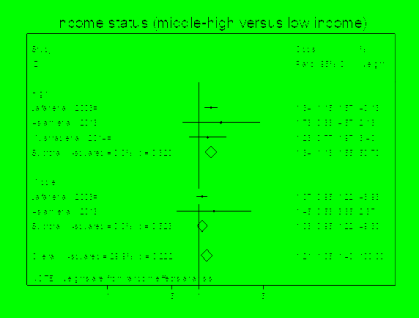
**

Supplement: S8 Fig — (DOCX) [file pone.0246085.s008.docx]

**S9 Fig : Funnel plots assessing publication bias in the results for income status**

**
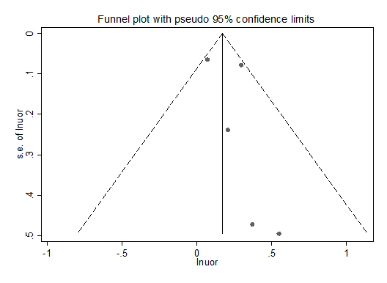
**

Supplement: S9 Fig — (DOCX) [file pone.0246085.s009.docx]

**S10 Fig : Forest plot from the Meta-analysis of physical activity (active versus sedentary)**

**
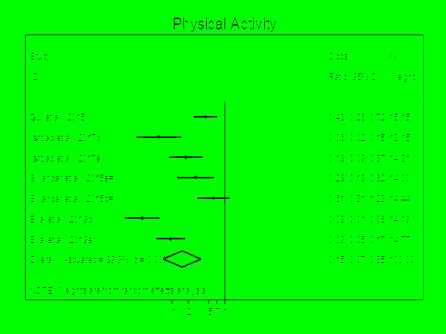
**

Supplement: S10 Fig — (DOCX) [file pone.0246085.s010.docx]

**S11 Fig : Funnel plots assessing publication bias in the results for physical activity**

**
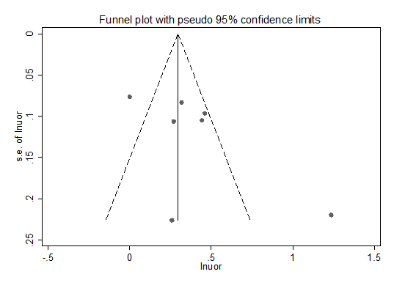
**

Supplement: S11 Fig — (DOCX) [file pone.0246085.s011.docx]

**S12 Fig : Forest plot from the Meta-analysis of unrestricted salt use**

**
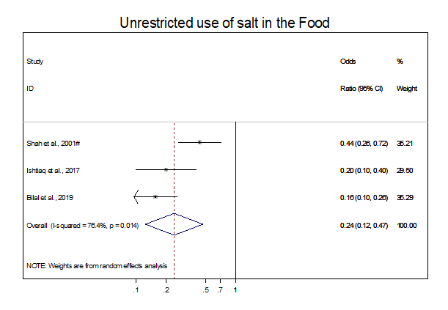
**

Supplement: S12 Fig — (DOCX) [file pone.0246085.s012.docx]

**S14 Fig : Forest plot from the Meta-analysis of tobacco use**

**
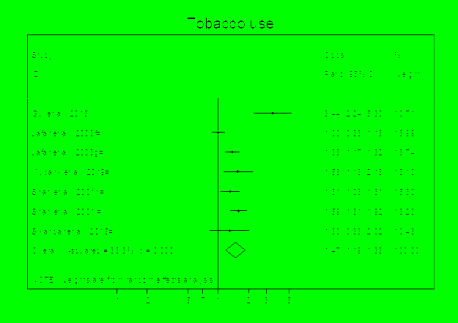
**

Supplement: S14 Fig — (DOCX) [file pone.0246085.s014.docx]

**S15 Fig : Funnel plots assessing publication bias in the results for tobacco use**

**
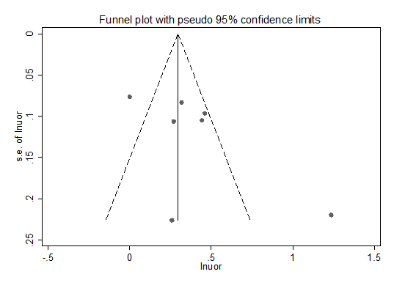
**

Supplement: S15 Fig — (DOCX) [file pone.0246085.s015.docx]

**S16 Fig: Forest plot from the Meta-analysis of family history of hypertension**

**
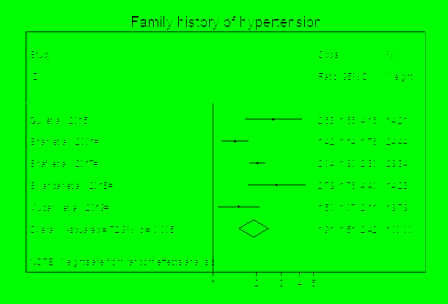
**

Supplement: S16 Fig — (DOCX) [file pone.0246085.s016.docx]

**S18 Fig : Forest plot from the Meta-analysis body mass index(BMI) groups**

**
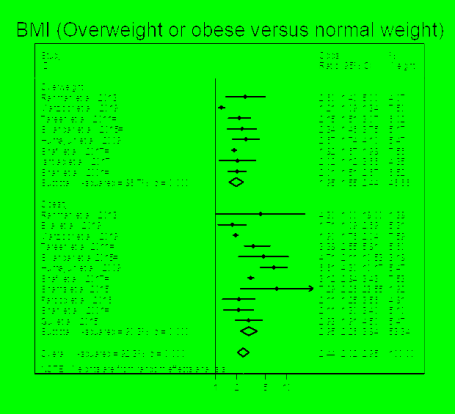
**

Supplement: S18 Fig — (DOCX) [file pone.0246085.s018.docx]

**S20 Fig : Forest plot from the Meta-analysis having diabetes**

**
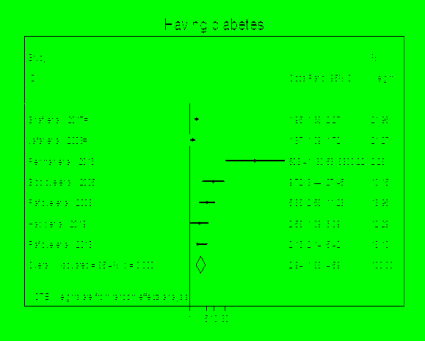
**

Supplement: S20 Fig — (DOCX) [file pone.0246085.s020.docx]

**S21 Fig : Funnel plots assessing publication bias in the results for having diabetes**

**
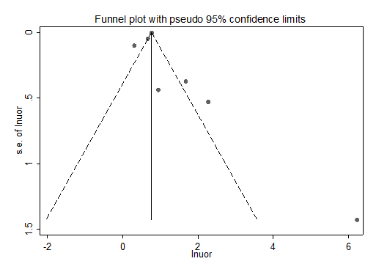
**

Supplement: S21 Fig — (DOCX) [file pone.0246085.s021.docx]

**S22 Fig : Forest plot from the Meta-analysis having anxiety**

**
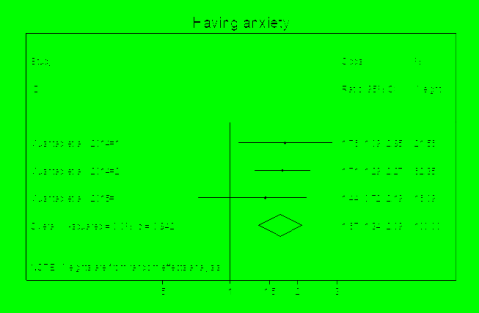
**

Supplement: S22 Fig — (DOCX) [file pone.0246085.s022.docx]

**S23 Fig : Funnel plots assessing publication bias in the results for having anxiety**

**
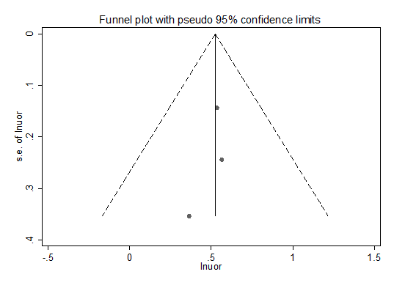
**

Supplement: S23 Fig — (DOCX) [file pone.0246085.s023.docx]

**S24 Fig : Forest plot from the Meta-analysis having stress**

**
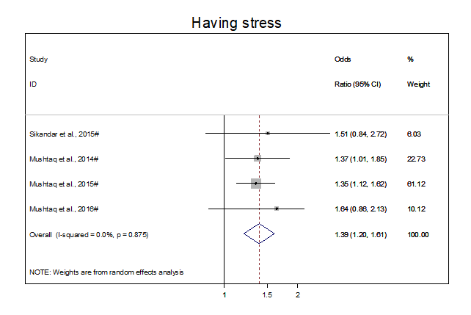
**

Supplement: S24 Fig — (DOCX) [file pone.0246085.s024.docx]

**S25 Fig Funnel plots assessing publication bias in the results for having stress**

**
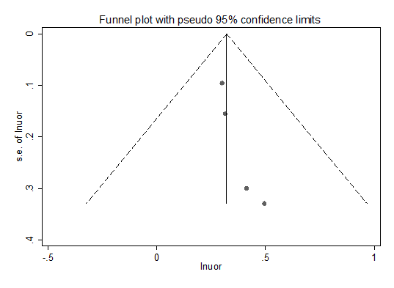
**

Supplement: S25 Fig — (DOCX) [file pone.0246085.s025.docx]

**S26 Fig : Forest plot from the Meta-analysis for anger-in (& -control)**

**
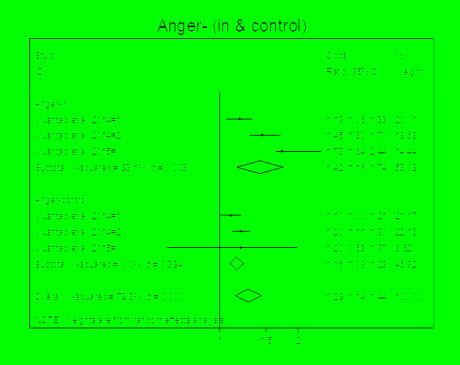
**

Supplement: S26 Fig — (DOCX) [file pone.0246085.s026.docx]
