# Supplementary material for: Factors associated with hypertension in Pakistan: A systematic review and meta-analysis
Source: PLoS One. 2021 Jan 29;16(1):e0246085. doi: 10.1371/journal.pone.0246085 (PMC7845984; doi:10.1371/journal.pone.0246085)
Supplement: S13 Fig — (DOCX) [file pone.0246085.s013.docx]

**S13 Fig : Funnel plots assessing publication bias in the results for unrestricted use of salt in the food**

**
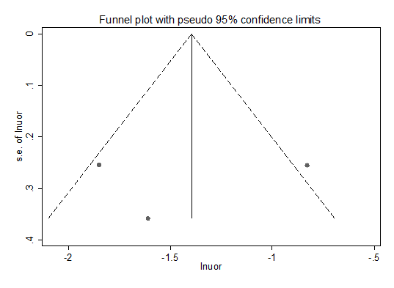
**
