# Supplementary material for: Factors associated with hypertension in Pakistan: A systematic review and meta-analysis
Source: PLoS One. 2021 Jan 29;16(1):e0246085. doi: 10.1371/journal.pone.0246085 (PMC7845984; doi:10.1371/journal.pone.0246085)
Supplement: S27 Fig — (DOCX) [file pone.0246085.s027.docx]

**S27 Fig : Funnel plots assessing publication bias in the results for anger-in (& -control)**

**27_1: anger-in**

**
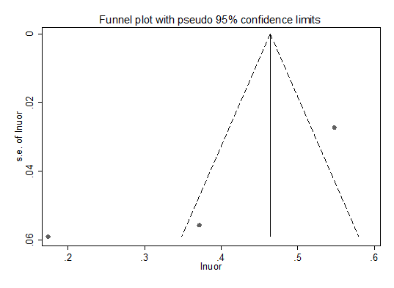
**

**27_2 anger-control**

**
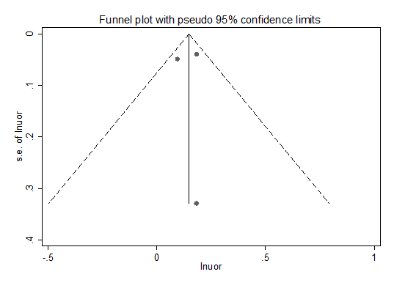
**
