# Supplementary material for: Factors associated with hypertension in Pakistan: A systematic review and meta-analysis
Source: PLoS One. 2021 Jan 29;16(1):e0246085. doi: 10.1371/journal.pone.0246085 (PMC7845984; doi:10.1371/journal.pone.0246085)
Supplement: S28 Fig — (DOCX) [file pone.0246085.s028.docx]

**Sensitivity Analyses:**

**S28 Fig : Forest plots for the meta-analyses of factors with effect estimates from three or more available high-quality studies (NOQAS sore ≥ 7).**

**28_1: sensitivity**

**
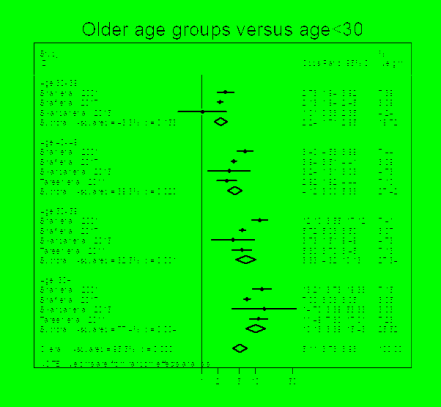
**

**28_2: sensitivity**

**
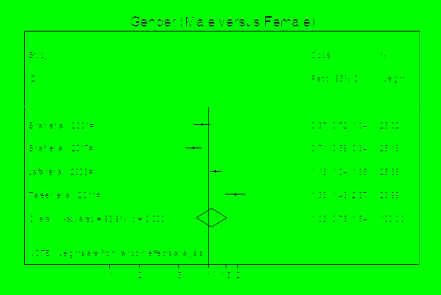
**

**28_3: sensitivity**

**
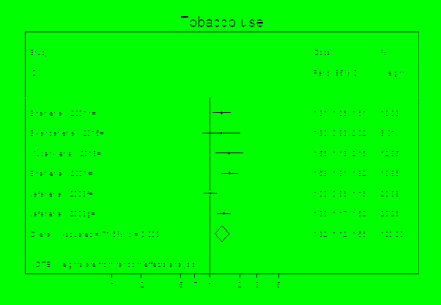
**

**28_4: sensitivity**

**
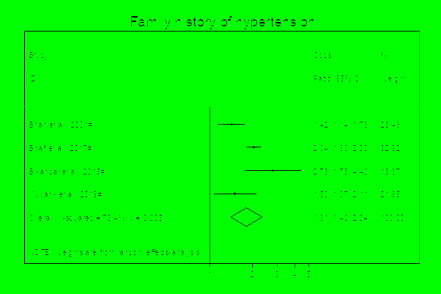
**

**28_5: sensitivity**

**
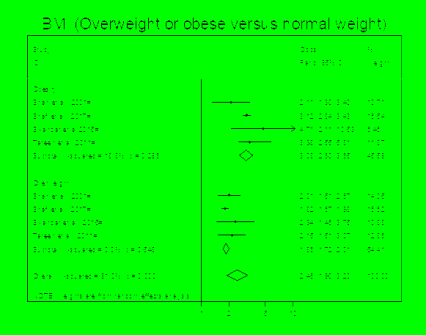
**
