# Supplementary material for: Factors associated with hypertension in Pakistan: A systematic review and meta-analysis
Source: PLoS One. 2021 Jan 29;16(1):e0246085. doi: 10.1371/journal.pone.0246085 (PMC7845984; doi:10.1371/journal.pone.0246085)
Supplement: S1 Table — (DOCX) [file pone.0246085.s029.docx]

S1 Table : Characteristics of studies included in the systematic review

| **Study Ref. No.** | **Author^a^,year, location** | **Methods** | | **Statistical methods** | **Participants characteristics and**  **Sample size^b^**  **sex, age** | **Measurement of BP and Hypertension,** | **Use of BP lowering medication** | **Major Comorbidities** | **Predictors examined**  **confounders controlled** | **Significant predictors of hypertension.**  **(Effect size: OR (95% CI)** | **^c^NOQAS** |
| --- | --- | --- | --- | --- | --- | --- | --- | --- | --- | --- | --- |
| 30 | Akatsu et al, 1996, Karachi | Aim: To assess the chronic health condition by estimating hypertension and obesity in women aged >25 years in squatter settlement of Karachi. Design: Community based cross- sectional study was carried out, Sector 8 (with average monthly income Rs. 1655) out of 12 sectors was chosen purposively. Trained investigators were hired to obtain data on weight, height, BP measurement (in two visits) and waist- hip measurements from the participants. | | Chi square test | 151 females aged over 25 living in Kachi Abadi of Chanesar Goth were included in this study. The maximum number of participants were observed in younger group 26- 35 while the mean age was 40 years. The mean weight was 54.3± 12.6 kg (ranged 26 to 105 Kg), mean height 153.5± 6 cm (ranged 126- 169 cm) and mean BMI was 23± 5.1 (ranging 12 to 44). | All the measurements were made by Community Health Workers (CHWs) according to the standard protocol. Hypertensives (**BP≥140/90 mm Hg or use of medication**) were categorized as with Mild HTN 16(34.8), Borderline Isolated Systolic HTN 9 (19.6) and anti- hypertensive drug taker 21 (45.6). The overall prevalence of hypertension was 46(30.5), higher prevalence was observed over 45 years of age. | 21 out of 46 hypertensives were anti- hypertensive drug taker. | NR | Women age- groups. | Increased prevalence of hypertension was observed among tobacco users, regular pan users, birth control pill users and overweigh /obese individuals. | 6 |
| 31 | Aslam et al, 2013, Karachi | Aim: To examine the relationship between diet and socioeconomic conditions and find out the prevalence of  stress and Hypertension in different socioeconomic classes.  Design: Community based cross- sectional study design. A self- structured questionnaire was used to collect base line data and to obtain three days dietary record of the participants. BP of each study subject was measured and was recorded on questionnaire. Stress was assessed using questionnaire adopted from International Stress Management Association UK. | | Kruskal Wallis H test, Chi- square and Spearman Correlation test. | This study was carried out between May 2010 to October 2011 among 176 participants of either gender, aged 20 to 60 years in Karachi. The mean age was 38.51± 11.06 years  . Out of 176, 54 were observed in High income Class, 36 in High middle-income group, 42 low middle-income group and below poverty group had 44 study subjects. | Participants were designated hypertensives if the average of two reading (10 min apart) **≥140/90 mm Hg.**  On BP examination, in below poverty group minimum percentage of population 18.2 (8 people) had hypertension. High income class and High middle- income group had same prevalence (15 (27.8%) and 10(27.8%)). 9 (21.4%) people had hypertension among the participants of low middle- income group. | NR | NR | Different socioeconomic groups with respect to income (high class, high middle-income group, low middle-income group, below poverty group). | High class and high middle-income group had higher prevalence of hypertension. | 5 |
| 32 | Aziz et al, 2005, Karachi | Aim: To estimate the BP profile (Prevalence, awareness and determinant) of the inhabitants of Metroville coloney Karchi and to make comparison with National Health Survey Pakistan and National Health and Nutrition Examination Survey USA.  Design: Metroville National Institute of CVD conducted a five- year prospective Metroville Health Study (MHS) in 1994. A convenient sample of 400 volunteer households were included to treatment and control groups randomly. Data on baseline characteristics was recorded and their BP, weight and height measurements were obtained. | | Multiple Regression Analysis | Data surveyed in Metroville Health study (MHS, 1994), Pakistan National Health Survey (PNHS 1990- 1994) and National Health and Nutrition Examination Survey (NHNES 1988- 1994) on adults of either gender, aged 18 years and above was included in this study. MHS had a sample of 946 adults (476 males and 470 females), PNHS had 6103 (males 3049 and females 3054) and NHNES had 16623 (males 7964, females 8659) participants aged 18 years or above. In MHS, the range of monthly income of per household was 1,500- 90,000. In MHS, 22% of participants were housewives, 36% were students, government servants were 5.6%, retired persons 1.7%, 8.3% other jobs and with no job description 7.3%. | Blood Pressure reading was obtained twice using sphygmomanometer, International standardized protocol was considered for the diagnoses (BP**≥140/90 mm Hg or use of BP- lowering medication**). The prevalence of HTN in MHS was 23.3 (220/946) among male 23.1 and female 23.4, in PNHS 15.9 (969/6103) male 17.3, female 14.5 and prevalence in NHNES was 25.4 (4229/16623), among male 26.0 and female 24.9. Of these three samples, Metroville was more prevalent to hypertension in all age- groups of men and women, for both sexes maximum number of hypertensives were seen in the age group 50- 75 in MHS, PNHS and NHNES samples. In hypertensives of all age group, females were more aware of their condition than men and therefore the rate of treatment was found high among females. By comparing the awareness and treatment in men, significant difference was observed in MHS versus NHNES (NHES men were more likely to be aware and treated). NHNES women and MHS women were more likely than PNHS to be aware of HTN. | In MHS, 27(24.5) men and 67(60.9) women were under treatment. In PNHS men on medication were 46(8.7), women 82 (18.6). MHS both sexes were more likely than PNHS to be treated (P-value <0.0001). In NHNES 925 (44.6) men and (62.5) women had treatment. NHNES men and women were more likely than MHS- men and women to be treated for hypertension (P- value <0.0001) | NR | Gender, age and BMI | Growing age and BMI were turned out significant factors of hypertension in all the three samples. | 6 |
| 33 | Bilal et al, 2019, Multan. | Aim: To determine the predictor of hypertension and to explore awareness among outpatients about their health status.  Design: cross- sectional study design was adopted, enrolled participants from Outpatients departments of four hospitals in Multan using systematic random sampling technique. Participants were interviewed their baseline characteristics, knowledge about hypertension and BP readings were recorded on a structured questionnaire. | | Descriptive statistics, Chi- Square test. | A total of 364 adults comprising 151 (41.5) males and 213 (58.5) females,  aged between 18 and 60 were participants of this study. 228 (62.6) of the participants had age group 18- 39, 164 (45.1) had secondary education, overweight was 173 (47.5), married 246 (67.6), 117 (32.1) were employed and  203 (55.8) had salt restricted diet. | Medical practitioners and trained nurses were employed to measure BP using mercury sphygmomanometer. The mean of three readings (obtained in single visit) were used in the analysis. According **to JNC VIII (BP > 140/ 90 mm Hg)** overall prevalence was 141 (38.7). Of these 122 (86.5) were aware of their medical status. The prevalence among females was  109 (77.3), 86 (60.9) among age group 40- 59, among overweight 85(60.3), married 111 (78.7), 99 (70.2) among unemployed and with use of salt restricted diet  114 (80.9). | 128 (90.8) were on medication for lowering BP. | NR | Gender, age, education, financial status, marital status, physical activity, smoking, weight and salt intake. | Gender, age, education, financial status, marital status, physical activity, smoking, weight and salt intake were significantly associated risk factors of hypertension. | 6 |
| 34 | Farooq et al, September 2016, Lahore Pakistan. | Aim: To investigate the prevalence and determinants of hypertension among businessmen in the city of Lahore  Design: Cross- sectional Community based study, using a structured questionnaire for data collection. | | Descriptive statistics. | 400 businessmen, 162 men and 238 women having their age between 20 to 45 years were recruited. | JNC VII guideline **(BP>= 140/90 or use of medication)** was adopted for diagnoses of hypertension.  Mercury Sphygmomanometer was used to measure BP. Prevalence of HTN was obtained 37.5% among all study subjects. | 32% (48) of hypertensives (150) were using BP lowering medicine. | NR | Smoking, use of red meat, BMI, Family history, Socio-economic status | Hypertension was associated with high salt intake and sedentary life style | **4** |
| 35 | Gul et al, 2015, Hayatabad Peshawar | Aim: To assess various risk factors for HTN in an urban adult population of KPK    Design: Cross- sectional Community based study, using a structured questionnaire for data collection | | **Chi-Square** | 500 working individuals of either gender, aged 20 to 70 years between April and October 2014, aged 20 t0 70 years), 353(70.6%) male, 26.6% smokers; 26.8% obese or overweight; family history of hypertension 20.6%. majority (89%) were age>30; 31.6% were checking BP regularly. | BP was measured using a manual mercury sphygmomanometer. BP? Prevalence of HTN **(BP≥140/90 mm Hg or use of medication for htn)** 128 (25.6%), males 87 (27.5%), female 41  (27.9%). | NR | NR | Age, Regular bp checking, marital status, type of diet, occupational status, BMI, Smoking, life style habit, family History | Underweight/ Normal weight, people who did not smoke, active life style, and having negative family history were less likely to be hypertensive. | 5 |
| 36 | Gupta et al, 2017, Agha Khan University Karachi, Pakistan. | Aim: To explore the socioeconomic factors associated with the prevalence, awareness and treatment of HTN among rural and urban dwellers of South Asian Countries, including Pakistan.  Design: For this study data was determined from a large- scale prospective cohort study PURE (Prospective Urban Rural Epidemiology 2003- 2009). Present study included data on household wealth index, socio economic and demographic factors followed by anthropometric, biochemical and BP measurements. Study participants were all eligible volunteers from 7 rural urban sites in Bangladesh, Pakistan and India. | | Logistic Regression Model. | This study was carried out on three South Asian Countries India (5 rural urban sites), Bangladesh (Dhaka) and Pakistan (Karachi) between the year 2003 and 2009. Consented participants aged 35- 75 from 150 community at 7 rural urban location were administered. Overall sample size for the study was 33, 433(Mean Age 48.3 ± 10.3 years) comprised 1,742 (5.2) participants from Pakistan with mean age 47.58± 8.9, 2934 (8.8) from Bangladesh with mean age 45.96± 9.3 and from India 28747 (86.0) with mean age 48.60 ±10.4. Overall male participants were 44.2% and female 55.8%. Overall rural dweller comprised 52.6% (17,577) of the study sample. Female participants at Pakistan, Bangladesh and India was 917 (52.6), 1,602 (54.6) and 16,135 (56.1) and subjects at rural sites are 762(43.7), 1,555 (53.0) and 15,260 (53.1) respectively. | BP of the study participants was measured thrice in sitting position with instrument Omron HEM-757 and average of two final readings was considered for analysis. Overall prevalence of HTN (BP**≥140/90 mm Hg or use of BP- lowering medication**) was 9,798 (31.6), comprised 434(24.9%), 1,080 (39.3) and 8,284 (30.7) in Pakistan, Bangladesh and India respectively.  Overall urban prevalence was higher than rural (unadjusted 38.7 vs 24.9, age-adjusted 38.6 vs 26.3) this difference was statistically significant. | Of those 9,798 (31.6) hypertensive, 31.9% were on treatment. In Pakistani- sample treated hypertensives were 135 (37%). | NR | Gender, age, rural/ urban study sites, wealth index, educational status and social capital index. | Growing age, high wealth index, lower social capital index, urban sites and greater education were significantly associated factors of hypertension. | 8 |
| 37 | Habib et al, 2019, district Mianwali, Punjab. | Aim: To investigate factors associated with elevated BP and different levels of hypertension. Design: Cross- sectional study design was employed that collected data from women working in different purposefully selected educational institutes of district Mianwali. All working women who answered fully to the designed questionnaire were included, their blood sample were taken, BMI and BP measurement were obtained. | | Descriptive statistics, Chi- Square test | Overall 262 female subjects aged 20 years or above working at educational institutes were investigated. Out of 262 subjects, 155 (59.2) were from Govt Schools, 17 (6.5) from Govt Colleges, private schools 75 (28.6) and 15 (5.7) working women were enrolled from private colleges. Participants were divided among three age groups, the maximum number of subjects 110 (42.0) were seen in age group 20- 30 years. A sample of 285 was selected for different clinical exposure, of which 38 (13.3) had diabetes, headache 168 (58.9), CVD 19 (6.7) and other diseases (body pain, blurry vision etc) 59 (20.7). | BP was measured three times on right arm and average of three readings were considered for the analysis. Overall prevalence of hypertension **(BP> 140/90)** was 142 (54.2). High prevalence 97 (62.6) was seen among women working in Govt schools. Prevalence of hypertension increased with an increase in age, participants in the age group 40 years and above had higher prevalence 51 (96.2). | NR | 22 (57.9) of the hypertensives had diabetes, 72 (42.8) had headache, CVD in 14 (73.7) and other disease (body pain, blurry vision etc) in 12(20.3) | Different educational institutes, age, weight, diabetes, headache, CVD, other disease (body pain, blurry vision etc), low socioeconomic status, low physical activity, urban population, high level of calcium, potassium and sodium. | Age, weight, low socioeconomic status, low physical activity, CVD, urban dwellers, high level of calcium, potassium and sodium were associated with the high prevalence of hypertension. | 6 |
| 38 | Humayun et al, 2009, Peshawar | Aim: To determine the relationship of hypertension with BMI and age (as the best predictors of HTN).  Design: cross- sectional observational study based on data collected using standard questionnaire and physical examination. | | Descriptive analysis | A total of 1006 adults (aged 20 years or above) of either gender, were recruited. 1006 study subjects comprised 541 (54%) males and 465 (46%) females, divided into three age groups:  Group I (20–39 years) 270 participants, Group II (40–59 years) 574 and Group III (≥60 years) 162 subjects.  Normal BMI 120(11.93),  Overweight 372 (36.98) and Obese 514 (51.09). | Average of two or more readings of BP, with an interval of two minutes were recorded from each randomly selected participant using mercury sphygmomanometer. Overall prevalence of HTN (BP≥140/90 mm Hg) was 655 (65%).  Prevalence among males was 340 (63%), female 315 (68%). | NR | **NR** | BMI, Age, gender | 41 (34.17%) of individuals with normal BMI had HTN, over half of Overweight 216 (58.06%) and 398 (77.43%) of Obese had hypertension.  Prevalence among age Group I (20–39 years) was 150 (56%), Group II (40–59 years) 395 (69%) and Group III (≥60 years) 110 (68%). | 5 |
| 39 | Ikramullah et al, 2014, Peshawar | Aim: To estimate correlation between body mass index and hypertension.  Design: This cross- sectional study was performed on data collected randomly from various occupational groups in Peshawar heart study (PHS). Recruited subjects were interviewed and their BP measurement and random blood sugar was recorded (No description of weight/ height measurement for BMI). | | Descriptive statistics, Pearson rank correlation | A total of 2548 subjects with mean age 37.4±11.5 years (range: 12- 85) comprising  1989(78.1%) males and 559(21.9%) females were recruited. Participants were divided into two groups obese 1540(60.4%) (with mean age 40.5±9.2) and non- obese 1015(39.6%) (Mean age 33.9±7.9 years) using BMI. Average weight, height and BMI of the total study population was 71.28±13.5 kg, 163.81±12.5 cm and 26.89±3.7  2 kg/m respectively. Non- obese group had mean SBP 120.8±32.7 mmHg and DBP 78.8±18.9 mmHg, obese group had mean SBP 130.7±38.2 mmHg and DBP 85.7±20.1 mmHg. | Mercury sphygmomanometer was used to measure BP in sitting position. JNC- 7 criteria **(SBP≥140 and/ or DBP≥ 90 or use of BP- lowering medication) =** was used to define hypertension. Out of total 2548 study population 28.26% (720) had  systolic BP equals or above 140 mmHg comprising 16.87% (170) non- obese subjects and 36.3% (555) obese. Total subjects with DBP 90 mm Hg and above were 0.94% (1043) that contained 25.7% (259) non- obese and obese 51.1% (787). | NR | Out of 1763 (69.19) hypertensive cases 1342 (52.67%) participants had obesity. | Obesity | Positive correlation had been obtained between hypertension and obesity with positive 0.2 Pearson rank correlation. | 6 |
| 40 | Ilyas et al, 1980, Peshawar | Aim: To explore the prevalence of hypertension among subgroup population of juvenile (5- 20 years of age) and adults (20- 60 years of age, active in service) in Peshawar, Pakistan.  Design: Community based cross- sectional design was adopted, data was collected on measurement of BP from different professional groups of adults and juvenile. | | Descriptive Statistics analysed survey data. | This population- survey was undertaken in Peshawar city between 1976 to 77. A sample of 7,103 based participants, 3,930 from juvenile aged 5 to 20 years and 3173 in- service adults of age 20 to 60 years were investigated. Juvenile group 3930 (55.3%) with 1656 males and 1681females composed of: 1656 (42%) school students, college students 1681(42.7%) and 593(15.1%) active in-Service. Sub- groups of in- service adults 3173 (44.7%) were: female teachers 398 (12.5), factory workers 902 (28.4), police force 800(52.2), male civil servant865 (27.3) commandos 208(6.5). | Methodological consideration and protocol of WHO  cooperation project 1973 was used to measure BP after resting for 20 minutes. Elevated BP among three BP readings were considered for analysis. In juvenile group prevalence of HTN (**BP≥160/90 mm Hg**) was 1.1% (5/ 1380) among students with age 5-10 years, 1.6% (32/1957) in students with 11-20 year and 1.5% (9/593) among in- service juvenile. Prevalence of HTN (**BP≥165/90 mm Hg**) among professional groups of adults were: 3.3% (13/398) female teachers, male factory workers 2.5% (23/902), male police force 4.5% (36/800), male civil servants 5.5% (48/865) and 1.9% (4/208) male commandos. | NR | NR | Adults professional groups, juvenile gender, juvenile school college students, juvenile professional groups | NR | 5 |
| 41 | Ishtiaq et al, 2017, Rawalpindi Islamabad, Pakistan | Aim: To determine the prevalence of hypertension and to explore the risk factors associated with it.  Design: The design of the study was Community based Cross- sectional. Data on basic characteristics, followed by BP and anthropometric measurements was recorded on a Structured questionnaire from randomly selected study sample. | | Chi Square Test | This community- based study was carried out over a period of six months (from March to August 2014). This Study incorporated 219 inhabitants of Rawalpindi- Islamabad having their age 18 years or greater, excluding study population experiencing pregnancy or other acute illness. Out of 219 individuals, Males were 89 (40.6%) and females 130(59.4%). Over half of the participants 110 (50.2%) had age group 18- 28 years, only 23 (10.5) had age above 60. Over half of the study sample 111(50.7%) was unemployed, employed 70 (32%), retired 17(7.8%) and students 21(9.6%).  This study observed 27(12.3%) as smokers, obese 103(47.1%) and 95(43.4%) individuals have low physical activity. 27 (42.2%) had family history of HTN, 43(67.2%) individuals revealed that high BP affects their day to day activities. | JNC-VIII guideline was used to diagnose HTN. Prevalence of hypertension **(BP≥140/90 or use of medication for htn)** was 64 (29.22%), | Out of 64(29.22%) hypertensives 59 (92.2%) were on BP lowering medication only 5 were newly diagnosed cases. | NR | Age, gender, occupation, education, weight, salt intake, family status and smoking. | Hypertension was directly associated with age, gender, family status, weight, educational level, economical status, physical activity and salt restricted diet. | 6 |
| 42 | Jafar et al, 2003, all four provinces of Pakistan. | Aim: To investigate the sub-group difference of hypertension Pakistani population.  Design: Cross-sectional study design was used. Pakistan Medical Research Council (PMRC) collected data with the help of US National Center for Health Statistics (NCHS) between 1990 and 1994. Two stage stratified sampling design was used with 80 rural and urban areas of four Provinces considered as primary sampling unit. Desired number of samples were recruited. | | ANOVA,Chi-square, Linear Regression model and Logistic Regression were used for the analysis. | A total of 8276 study subjects aged 15 years were included from the third National Health and Nutrition Examination Survey (NHANES III) conducted over a period of four years. Subjects from different ethnic subgroups of Pakistani population were investigated. Ethnicity of the participant was defined on the bases of their mother tongue. Among 8273 participants 1504 were Sindhi, Balochi 293, Pashtun 1138, Punjabi 3669 and Muhajir were 1669. Population study consist of 3927(47.4) Males and 4349(52.5) Female with their mean age 36.2 (17.1). Urban dwellers 3197, literate 2850, diabetic 444, cigarette smokers 1277 and participants with low socio- economic status were 2501. Mean BMI was observed 21.3 (5.0). | Blood test BP and anthropometric measurements were obtained from each selected subject. Subjects were considered hypertensive if the average of multiple reading was ≥140/90 or if they were using antihypertensive medicine. The overall prevalence was 19.0% (18.9-19.1%) with Age- adjusted prevalence in men was ranged from 17.3 to 25.3 % and 9.9 to 41.4% in female, among five ethnic subgroups. | NR | Study population consisted 444 diabetic subjects, among those 174(39.2) were hypertensive. | Ethnic groups, Gender, urban/ rural differences, literacy, BMI, diabetes, Tobacco use, High intake of meat, daily intake of ghee, daily intake of fruit and daily intake of Calcium food. | **Variable: UAOR (95% CI), AOR (95% CI)**  Ethnicity Muhajir: 1.83(1.53-2.21), 1.37(1.10-1.69)  Punjabi: 1.28(1.08-1.51), 1.05(0.88-1.27)  Pashtun: 2.29(1.88-2.77),1.91(1.52-2.39)  Balochi: 2.92(2.20-3.89),2.71(1.97-3.75)  Sindhi: 1.0, 1.0  **Sex**- Male: 1.19 (1.06-1.32), 1.18(1.04- 1.35)  Urban: 1.34(1.20-1.49), 1.03(0.89-1.19)  Literate: 1.04(0.93-1.17), 0.80(0.69-0.93) and  Diabetes: 2.79(2.29-3.41), 1.37(1.09-1.72) had significant association with hypertension. | **8** |
| 43 | Jawed et al, 2017, Faisalabad. | **Aim:** To estimate the prevalence of different blood groups and to investigate BMI and blood groups as a risk factors of hypertension.  Design: It was a cross- sectional survey conducted on female medical students of Faisal Abad. Female medicine students were selected by multi- stage random sampling from the total students of Faisal Abad University. A questionnaire was administered to collect all the relevant data and to record their Physical and BP measurements. | | Mantel-Haenszel (MH) age-adjusted odd ratios (ORs), multiple regression analysis and logistic regression. | This study was carried out on 145 female students between March and April 2016. Age of the participants ranged between 17 and 23 year and mean age was 18.4 years. Of these 145 subjects 27(18.6%) had blood group A, B 65(44.8%), 43(29.7%) had O and the proportion of group AB was 10(6.9%). Participants with blood group A had higher mean SBP 118.9± 13.5 and DBP 81.3 ± 8.2 as compared to other groups. A high proportion of sample had Rh- positive 130(89.6%) of these 32(24.6%) had pre- hypertension and Rh- negative was 15(10.4%) with 1(6.6%) pre- hypertensive. The study sample had mean BMI 227.62kg/m2, where mean weight was 58.1 ± 6.3kg and mean height 1.69 ±1.20 metres. | Blood pressure of the participants were measured after relaxing them for few minutes. mercury sphygmomanometer with a proper cuff size was used for measuring BP. Joint National Committee guideline- VIII was used for detection of pre- hypertension (121/81 to 139/89 mmHg) and hypertension **(BP > 140/ 90 mm Hg)**. Average value of SBP and DBP was 113.1± 2.6 and 77.9± 06. | NR | NR | Blood groups, BMI | Blood group_ O was significantly associated with DBP (OR 5.05), BMI was found associated with both SBP and DBP with P- value 0.004 and 0.042. | 8 |
| 44 | Khan et al, 2012, Peshawar. | Aim: To investigate the prevalence of hypertension among adults who had migraine or tension headache.  Design: Cross- sectional community-based design was employed. Participants were all visitors (during study period) of a private clinic presenting with headache. Participants were examined clinically; their headache were classified as migraine or non- migraine and data on BP measurement was obtained. (data collection tool not reported) | | Descriptive statistics | A total of 972 adult subject presenting with headache were enrolled between January 2002 and December 2003. Participants were categorized as having migraine headache 261(26.8) or non- migraine headache 711 (73.1) as per protocol of international society of headache. Of 261 migraine patients 15(5.75) had hypertension and there were 180 (25.3) non migraine hypertensive patients. | Blood pressure was measured by a mercury sphygmomanometer (no description of measurement procedure reported). JNC- VII **(BP > 140/ 90 mm Hg)** guideline was used for diagnoses of hypertension. Prevalence of hypertension was 195 (20.06), of these 112 (11.52) had stage- I and remaining 83 (8.53) had stage- II hypertension. | NR | NR | headache | OR (95% C- I)  Headache: 0.1799 (0.104- 0.3112) | 6 |
| 45 | Khan et al, 2015, Khyber Pakhtunkhwa | Aim: To investigate the risk factors of cardiovascular disease (CVD) in rural areas of Khyber Pakhtunkhwa. Design: This was a cross- sectional study. For data collection district health authorities and Pak Army deploy unit organised free medical camps. Data on life style and medical history were recorded on questionnaire from each volunteer visitor. Biochemical measurements (random blood sugar, cholesterol, BP, weight/ height) were also obtained. | | Descriptive Statistics | A total of 2569 visitors either male or female aged 25 to 64 (mean age 45.97 ±12.1) year were included in the study. Out of total study sample 1,314 (51.1) were males, most of the males 532 (20.7) were from age- group 55- 64 years. Females were 1255 (48.9), majority 348(13.5) had age- group 55- 64 years. 293 (11.4%) of the participants had diabetes, 937(36.5%) were obese, 607 (23.6%) had sedentary life style and 306 (11.9%) had positive family history of CVD. | Study subjects were defined hypertensive if they had SBP ≥140 mm Hg and or DBP ≥90 or previously diagnosed hypertension. Overall prevalence of hypertension was 686 (26.7), among male 372 (14.4%) and female 314(12.2%). 2,284 (88.9) had previous history of hypertension while remining 285 (11.1) were newly diagnosed cased. SBP ranged between 80 and 250 with mean value 128.91 ± 27.82 and DBP ranged 60- 160 with mean value 81.76 ±10.1 | 2,284 (88.9) had previous history of hypertension | NR | Gender, Age group | NR | 6 |
| 46 | Malik et al, 2013, Lahore | Aim: To determine the frequency of dyslipidaemia in non- obese and non- diabetic hypertensive patients in comparison to non- obese and non- diabetic normotensives.  Design: This was a case- control study conducted in services hospital Lahore using purposive sampling techniques. Data on baseline characteristics was obtained on questionnaire. Data on BP measurement was recorded, and blood samples were taken from each selected subject (with 10 hour fast) for biochemical analysis. | | t-test, Chi-square test.  Descriptive statistics and odd ratios. | This case- control study was conducted in Medicine Department of Lahore Services hospital from 2008 to 2009. A total of 120 individuals of age- range 35 to 65 years were investigated. Out of 120 subjects 40 were non- obese non- diabetic hypertensives and the remaining 80 were non- obese non- diabetic healthy controls. Individuals with systemic illness or those on medication for lipids control were excluded from study sample. The average age was 46.43 ± 7.58 and 46.96 ± 7.76 years for cases and controls. Both the groups had approximately equal mean value of FBS (88.83 ± 3.87 vs 89.80 ± 3.95) and BMI (23.38 ± 1.20 vs 23.66 ± 0.98). | BP of all individuals, including cases and controls was measured multiple times on at least two visits a and recorded on questionnaire. Among cases mean SBP and mean DBP was 158.01 ± 9.20 and 102.55 ± 4.8 mm Hg whereas among controls it was 121.04 ± 6.86 and 79.60 ± 6.38 mm Hg. | NR | NR | Age, gender, smoking, BMI, fasting blood glucose, dietary habits, lipid profile (total cholesterol, LDL- C, HDL-C and triglyceride) and lipid abnormality. | Lipid Abnormality:  TC (OR 2.96 CI 1.2- 7.4), LDL- C (OR 2.67 CI 1.12- 6.41), HDL- C in men only (OR 4.28 CI 1.35- 3.89) and triglyceride (OR 4.57 CI 1.89- 1.22) were statistically significant associated with hypertension. | 6 |
| 47 | Manzoor et al, 2019, district Faisalabad Punjab | Aim: To determine whether obesity and overweigh are associated factors for hypertension.  Design: Design of the study was cross- sectional community based, collected data from 45 health care centres of district Faisalabad using convenience sampling. Participants were screened for BP and anthropometric measurement between 19 and 24 February 2018. | | Chi Square test | A total of 43,943 participants of either gender aged 25 years or greater (mean age 43.9±12.6 years) were enrolled over a period of 6 days only (between 19 and 24 February 2018). The frequency of male and female subject was 13733 and 30210 respectively. Maximum number of participants 19231 (43.8%) were observed in age group 25- 40 years.  On the basis of BMI participants were categorized as normal (12,308 (28.1)), overweight (6536 (14.9)) and obese (25,030 (57)). Average weight and height were 67.28±14.39 kg and (1.59±0.09)m. | Study subjects were screened for BP and were considered as hypertensive if their SBP >= 140 mm Hg also DBP >= 90 mm Hg. The overall prevalence was 5320 (12.12), males 31.3% and females 68.7%. prevalence among overweight and obese was  644 (12.1%) and  3,657 (68.7%) respectively. | NR | NR | Gender, overweight and obesity. | BMI was strongly associated factor for hypertension. | 6 |
| 48 | Mubarik et al, 2019, Rawalpindi | Aim: To investigate the association of various demographic, health, social and physical factors with hypertension.  Design: A case- control study design The baseline data was collected by the staff of the hospital through structure questionnaire while BP measurements were obtained by the attending physician. | | Fisher Exact test and logistic Regression | This study was conducted between December 2016 to July 2017 at three hospitals of Rawalpindi. Survey included 2000 adult individuals, comprising 549 (27.45%) hypertensives and 1451 (72.55%) non hypertensives. Among hypertensive group the mean age was 43.32± 9.7 (ranged 22- 60) years and 31.8± 10.1 (15- 60) years was the mean age of control group. Among 549 hypertensives 482 (87.8) and 645 (44.5) among control were above 30 years of age. Age (>30: 87.8% of cases, 44.5% of control). | 549 (27.45%) patients with **BP≥140/90 mm Hg** and anti- hypertensive drug taker were designated as hypertensive cases. Attending physician confirmed all cases by measuring their BP. | 549 (27.45%) out of 2000 study subject were confirmed cases of HTN and were using anti- hypertensive medicine. | Hypertensive group had kidney disease 157(26.6), diabetes 227(41.3) and smokers 288(52.5). | Age, educational status, family history, smoking, diabetes and kidney diseases | Age (AOR 1.21, CI 1.19-1.24), smoking (AOR 1.56, CI 1.13- 2.16), kidney disease (AOR 2.75, CI 1.8- 4.2), diabetes (AOR 1.49, CI 1.05- 2.12), family history (AOR 1.5, CI 1.07- 2.11) and educational status (AOR 0.54, CI 0.39- 0.76) were significantly associated factors of hypertension. | 7 |
| 49 | Mushtaq et al, 2014, | Aims: To determine psychological predictors of HTN.  Design: Matched case-control design was used. Two groups of participants were selected, a hypertensive group and group of healthy controls. Data were collected using a structured questionnaire for demographic data, urdu version of Depression, Anxiety and Stress Scale (DASS) were administered for assessing psychological distress and data on component of anger from each individuals were obtained using urdu language State Trait Anger Expression Inventory (STAXI). | | The linear association between psychological variable and HTN was tested using Mentle Haenzel Chi- Square test. Binary Logistic Regression Model was used to find significant predictors of HTN and differences on psychological variables was tested by Independent sample t test. | **A** sample of 360 individuals, comprising hypertensive patients (216) and non-cases (144) both with the same age group, gender, working hours (4-8 hr) and monthly income (5000- 85000), was taken by purposive sampling technique. The required age range for the participants was 30-60 years. The mean age of hypertensive cases was 47.00 (SD = 8.27), mean monthly income 28236.11 (SD = 13891.76), mean working hours 8.63 (SD = 4.14), mean weigh 76.91 (SD = 8.63) and 188(87%) of the participants were from joint family system. Non hypertensive group was observed with the mean age of 43.00(SD = 8.10), mean monthly income 37881.94 (SD = 21002.32), mean working hours 7.86 (SD = 3.35), mean weight 72.71 (SD = 10.35) and 51 (35%) of the individuals were belonging joint family system.  Depression with mean score 28.78 (15.98), Anxiety 22.48 (10.90), Stress 32.27 (16.00) and overall psychological distress 82.18 (41.85). The mean score of these variables were as: state anger State anger 15.78 (6.80), Anger-in 17.78 (6.80), Anger-control 19.43 (9.68), Anger-expression 29.60 (7.64), Anger 115.88 (35.78). | Those patients’ visitors who had a confirmed diagnosis of hypertension, were taken for the study along with a healthy control group from the visitors of the same hospitals. | NR | NR | Depression, anxiety, stress, state anger, trait anger, anger in, anger out, anger control and anger expression. | Depression (OR 1.64, CI 1.37- 2.22), Anxiety (OR 1.71, CI 1.29- 1.4), Stress (OR 1.35, CI 1.12- 1.62), DASS (OR 1.4, CI 1.23- 1.58), State anger (OR 1.42, CI 1.25- 1.66), Anger- in (OR 1.45, CI 1.3- 1.7), Anger Control (OR 1.2, CI 1.11- 1.3), Anger Expression (OR 5.73, CI 0.98- 12.35), Anger Total (OR 1.08, CI 1.05- 1.11) had significant association with hypertension. | 7 |
| 50 | Mushtaq et al, 2014, Lahore | Aims: To investigate the correlation of depression, anxiety, stress and demographic factors with hypertension. .  Design: Matched cases (hypertensive)-control (normotensive) design selected by purposive sampling technique from two public hospitals of Lahore. Demographic information was obtained from selected subject using a structured questionnaire and a standard protocol, DASS (Depression, Anxiety and Stress Scale) was administered for measuring states of depression. | | Mentle Haenzel Chi- Square test and binary logistic regression model was used for data analysis. | A sample of 137hypertensive cases and their age- matched 100 Normotensive (Non-blood relatives of selected hypertensive cases) individuals were recruited. The required age for the participants was between 30 to 65 years. Out of 137 cases men were 77(56) and females 60(44) with 130 had a positive family of hypertension and only 49 had their spouse on job. Normotensive group (100) consisted 50 men and 50 women with only 6 had family history of hypertension and 47 participants had their spouse on job. Mean score of variable Depression was 15.78(11.49), Anxiety 20.62(13.94), Stress 21.42 (11.10) and DASS 57.62(32.91). | Patients already diagnosed with hypertension (those who are currently on antihypertensive medication) were taken for this study. | 137 patients who were on BP lowering medication were recruited in this study. | Patients with history of chronic diseases included diabetes, heart diseases, cancer, renal problem, liver problems and malignant were excluded from the study. | Gender, occupation, family history of hypertension, office job, monthly income, number of dependent, weight, working hours, spouse job, depression, anxiety, stress and DASS | Number of dependents (OR 1.42, CI 0.74- 1.85), Working Hours (OR 1.56, CI 1.03-2.27), Depression (OR 1.44, CI 1.10-1.88), Anxiety (OR 1.76 CI 1.09-2.89) and Stress (OR 1.37, CI 1.01-1.85) turned out significant predictors of hypertension. | 8 |
| 51 | Mushtaq et al, 2014, Lahore Punjab | Aims: To demonstrate the association of anger and its dimensions with hypertension.  Design: Matched Case- control design was employed to compare hypertensive and normotensive groups with respect to all dimensions of anger. Both the groups (hypertensive and Normotensive) were selected by purposive sampling technique from two outdoor public hospitals of Lahore. Demographic characteristics of the participants were recorded on questionnaire whereas component of anger was assessed using urdu version of State Trait- anger Expression Inventory (STAXI). | | Mentle Haenzel Chi- Square test and binary logistic regression model was used for data analysis. | 137 hypertensive cases and 100 healthy controls of the same age group, gender, working hours and income as hypertensives were selected for the study. The required age rang chosen for the study was 30 to 65 years. Out of 137 hypertensive cases (mean age 45, SD 8.37) males were 77(56%), females 60(44%), matriculate 70(51%), on job 64(47%), with family history of HTN 121(88%) and only 18(13%) were not living in join family system. Control group of 100 participants was composed of 50 males and 50 females with the mean age of 46(8.86). Family history of HTN was observed with only 10 and only 15 participants reported that they live in joint family system. | Patients diagnosed with HTN, who were currently on BP lowering medication were taken for this study. | 137 out of 237 | NR | Gender, education, occupation, new in city, family history of HTN, family system, component of anger: state- anger, trait- anger, anger- in, anger- control, anger- expression. | State- anger (OR 1.27, CI 1.09- 1.47),  Trait anger (OR 1.16, CI 1.2- 1.32), Anger- in (OR 1.19, CI 1.06- 1.33) Anger control (OR 1.1, CI 1- 1.21)  Anger expression (OR 1.85, CI 1.27- 2.69) had significant association with hypertension. | 8 |
| 52 | Mushtaq et al, 2015, Lahore | Aims: To determine whether the psychological states (depression, anxiety, stress and anger) of an individual predict hypertension.  Design: Matched case-control design was used for which two group of participants, cases (hypertensives) and control (normotensives) were selected from the same public hospitals by purposive sampling technique. Data on demographic information was collected by a structure questionnaire. Data on psychological states were explored using Urdu translated self- report Inventory, Depression Anxiety and Stress Scale (DASS) and State Trait Anger Expression Inventory (STAXI). | | Descriptive statistics, chi- square test and logistic regression | A sample of 270 participants from two public hospital was taken for this study. Out of 270 participants 200 (males 110, females 90) were confirmed hypertensive patient who were on BP lowering medication and 170 (males 90, females 80) were non hypertensive visitors of the same hospital who had no parental history of hypertension. Individuals with renal problem, heart patients and pregnant women were excluded from study population. `The required age range for both the group was 35 to 65 years (Mean = 44.34; SD = 8.32). Working hours ranged between 5 to 15 hours (M = 7.91; SD = 4.10), monthly income ranged 17550 to 72330 Rs (Mean = 30543; D=16454.12), weight was ranged 65 to 95 kg (M = 73; SD = 8.02) and the number of dependent family members ranged between 0 to 9 persons. The mean score of the psychological variables was: state anger 16.44 (6.66), Trait anger 22.20 (8.76), Anger-in 18.53 (8.13), Anger-out 14.67 (5.12), Anger-control 21.38 (8.17), Anger expression  25.56 (8.16), Anger 114.27 (38.12), Anxiety 23.83 (12.66), Stress 32.62 (15.12). | Out of 270 study subjects 200 patients with diagnosed hypertension, taking BP lowering medicine were selected for this study. | 200 out of 270 were on medication. | NR | Age, weight, working hours, monthly income, number of dependent family members, Psychological var: State anger, Trait anger, Anger-in, Anger-out, Anger-control, Anger, Anxiety, stress. | Anger- control (OR 1.2, CI 0.63-1.92), Anger- in (OR 1.73, CI 1.64-2.42), Anxiety (OR 1.44, CI 0.72-2.15), Stress (OR 1.64, CI 0.86- 2.13) turned out significant predictors of hypertension. | 7 |
| 53 | Nawaz et al, 2010, Lahore Pakistan. | Aim: To investigates the relationship of different sound levels with hypertension and prehypertension in Pakistani population.  Design: A cross- sectional study was undertaken in different working sites of Lahore and Jhang with sound pressure level ranges: equal or below 80 dBA (Normal sound), within 91- 84 dBA (Median Noise) and equal or more than 95 dBA (High Noise). | | T- test, Chi- Square test and Odd Rations were used for data analysis. | The study was conducted between November 2005 to January 2007. Participants were required at least 8 hour/ day exposure to specific sound level for continuously five or more years. A sample size of 389, with 154 participants from sites with normal sound intensity (teachers and students), 112 Medium Noise Sites (male drivers and conductors), and 123 participants from High Noise Sites (factory workers) were incorporated in this study. The required age range for the participants was 30 to 50 years. Participants with systemic chronic diseases were excluded from study sample. | To measure BP, Individuals were approached three times between 8am to 11 am. BP was estimated after taking three reading on each visit. Among 154participants exposed to normal sound level 15(9.7) were hypertensive (**BP≥140/90 mm Hg**) and 16(10.4) were pre-hypertensive (**SBP 121- 139 mmHg DBP 181-189 mmHg**). In sites with median sound level: hypertensives 18(16.1) with mean age 42± 5, Mean BMI 29± 3, Smokers 5/18 and family history 6/18. High noise group had 30(24.4) prevalence of hypertension with Mean Age 37 ± 6, Mean BMI 25± 4, Smokers 16/30 and family history 16/30. Prevalence of prehypertension among median sound group was 25(22.3) and 29(23.6) among sites with high sound level. | NR | NR | Occupational Noise Level, Age in Years, Weight, Height, BMI, Current Smoker, Family History. | Participants with median sound level (AOR 2.271, CI 1.043-4.946) and high sound level (AOR 4.41, CI 2.123-9.196) were significantly more likely to be hypertensive. | 7 |
| 54 | Rafique et al, 2002, Karachi | Aim: To investigate the prevalence and awareness of diabetes and hypertension and to determine the life style determinants of these medical conditions.  Design: Analytical cross- sectional study design to survey adult individuals attending one day health camp for awareness of diabetes at Agha Khan University Hospital(AKUH) Karachi. A structure questionnaire was used for collecting data on the baseline characteristics of individuals, measurement of weight, height, BP and Random Glucose reading was also recorded on questionnaire by the physician. | | Chi- Square test and T- test. | Among 264 adult individuals. Of these, Male participants were 141 and females were 123, with mean (SD) age 38 (12) years. Maximum number of participants 114 (43.2) were examined in age group 15- 34 while only 32(12.1) had age above 50 years. Participants had high literacy rate 160(60.6), 5 (1.8) had no formal education. Positive family history of diabetes, hypertension and both diabetes and hypertension were seen in 28 (10.6), 51 (19.3) and 101 (38.3) respectively. Previously diagnosed hypertensive cases were 24 (9.1), newly diagnosed were 39 (14.8). The proportion of current smokers was 19 (7.2) and past smokers 20 (7.6). Only 38 (14.4) participants had sufficient physical activity, exercising 2-4 times a week. Mean BMI among male and female was 25.4 ± 4.5 and 26.9 ± 5.3. | A physician measured BP using standard mercury sphygmomanometer. Joint National Committee guideline- vii (BP**≥140/90 mm Hg**) was adopted for the diagnoses of Hypertension (HTN). Overall prevalence of HTN in the study was 23.9% (63). Prevalence among males was 24.1% (34/141) and females was 23.6% (29/123). | NR | Over half of the diabetic cases had hypertension 55.6 (20/36). | Age, gender, BMI, blood glucose level | Increasing age and BMI had significant association with hypertension and diabetes (only P- value is reported). | 6 |
| 55 | Rafique et al, 2019, Islamabad | Aim: To estimate whether Leptin is a risk factor for hypertension or not.  Design: The case- control study design was adopted that recruited subjects in two groups (hypertensive cases and healthy controls) by using convenience sampling technique. Data on baseline characteristic, physical measurement (BMI), laboratory tests (FBS, Leptin, BP, Lipids) was recorded from both the groups after obtaining their written consent. | | Descriptive statistics, univariate and multivariable logistic regression. | A total of 104 males, comprising 52 hypertensive patients and 52 healthy controls were enrolled from the same college and hospital (Shifa college of medicine and hospital) in period of six months. Hypertensive group had higher mean value for BMI (28.01±0.12 vs 24.11±0.27), FBS mg/ dl (169.20±7.34 vs 127.50±2.30), SBP (168±7 vs 119±7), DBP (93±5 vs 75±4), leptin level ng/ ml (53.40±4.34 vs 21.65±3.50). Hypertensive group also had higher percentage of diabetics (42 vs 17), Hyperglycemics (24 vs 8) and Hyperleptinemics (75 vs 18) as compared to control group. | Medical technicians were employed to measure BP three times on both arms using a mercury sphygmomanometer. Subjects were considered in case_ group if they were found using BP lowering medicine or if their BP was greater than 140/ 90 mm Hg. Over all 52 male subjects were included in hypertensive group. | NR | Hypertensive group had higher percentage of diabetics (42), Hyperglycemics (24) and Hyperleptinemics (75). | BMI, leptin, cholesterol, FBS, Hyperglycemics, Hyperleptinemics | Variable: AOR (C-I)  FBS: 2.16 (2.14-5.42), Leptin: 14.5 (5.01- 35.6) had significant association with hypertension. | 8 |
| 56 | Rahman et al, 2013, Karachi. | Aim: To estimate the prevalence of elevated blood pressure and to determine its association with BMI, Haematuria and Proteinuria among school children.  Design: Cross- sectional study design was employed. Data on weight, height and BP measurements was obtained and urine dipstick was performed for detecting hematuria on school children. (No description of sampling design and data collection tool) | | Descriptive statistics, multiple logistic regression | Over all 661Public school children with the mean age of 14  ± 1.3 years were included in the study. Participants were classified as normotensive (18.8%), pre- hypertensive (15%) and hypertensive (3%). 602 (91.1) of the children were underweight, overweight and obese were 51 (7.7) and 8 (1.2) respectively. 18.5 ± 4.3  Kg/m2 was the mean BMI value. Urine dipstick for proteinuria was positive in 31 (4.7) and asymptomatic hematuria was detected in 8 (1.2). | Trained personnel were employed to measure BP using mercury sphygmomanometer. BP was measured on right arm in sitting position. Average of three readings was included in analysis. Prevalence of hypertension **(BP>= 95 centile, US normative table)** was 20 (3.0). prevalence of htn among overweigh was 6 (11.8) and 1 (12.5) in obese. | NR | NR | Age, gender, BMI, proteinuria, hematuria. | Variable: Relative Risk (C.I)  Age: 1.2 (1- 1.5)  Sex: 2 (1- 4.4) Proteinuria: 2.3 (7- 7.7)  Hematuria: 1 (2- 8.3) had significant association of hypertension. | 6 |
| 57 | Raza et al, 2019, Karachi. | Aim: To determine the prevalence of hypertension and its relation to the dietary habits among the working women and housewives.  Design: The design of the study was cross- sectional collected data from working women and housewives using convenient sampling. Demographic data and data on dietary habits followed by weight, height and BP measurement was recorded on questionnaire from each selected subject. | | Chi Square Test | This study enrolled 600 female subjects (aged 35 to 45) from the middle socioeconomic background. Of these 300 were housewives with at least 14 years of education and remaining 300 were working women of Karachi University and Dow University of Health Sciences, Karachi (DUHS). Housewives had average of 35.56 ± 7.53 years and 35.44 ± 6.65 years among working women. Housewives 204 (68%) had higher prevalence of hypertension as compared to working women 102 (34%). Of 204 hypertensive housewives 90 (44.1%) were overweight while 16 (15.7%) were overweight among hypertensive working women. Comparing the dietary practices among hypertensives revealed that more of the housewives were hypertensives than working women (vegetables per days: 86 (70.5) vs 32 (27.1), meal per day: 58 (56.9) vs 4 (20.0), fruits per day: 116 (69.9) vs 20 (25.6)). | Mercury sphygmomanometer was used to measure BP twice with an interval of half an hour to get accurate result. Reference range of BP for hypertension had not been reported in this study though hypertension prevalence among housewives was much higher 204 (68%) than working women 102 (34%). | NR | 48 (23.5%) of obese housewives had hypertension. | Housewives, working women, BMI, dietary and life style practices. | Being housewife, BMI, meal consumption per day, fruits consumption per day, fish/ poultry had significant association with hypertension. | 6 |
| 58 | Rehman et al, July 2016, Peshawar | Aim: To determine the prevalence of diabetes and HTN among old urban population and its association with financial dependency, sex and literacy  Design: Cross- sectional study design was used. Data was collected using semi-structure questionnaire and simple random sampling technique | | Chi square | This study investigated 250 geriatric people (>60 years) between April and May 2014. Study population contained 151(60.4) males, 99 (39.6) females, 145 (58%) literate, 105 (42%) illiterate and 93 (37.2) financially dependent on family 32 (12.8) partially dependent and 125 (50%) independent. | For the diagnoses of HTN and diabetes a self- report method or use of medication was considered instead of standard diagnostic tests. Prevalence of diabetes and HTN was 80(32%) and 121(48.4%). | NR | 80(66.11%) diabetic subjects were hypertensive | Sex, literacy, financial dependency on family, diabetes | Sex, literacy, financial dependency and diabetes were observed as associated factors of hypertension. | 5 |
| 59 | Safdar et al, 2004, Karachi | Aim: To estimate the prevalence of hypertension in a population of low- income settlement of Karachi.  Design: Cross- sectional, survey using multistage random sampling from ethnically mixed community with low- income. Study subjects were interviewed, and their BP was recorded by medical students. | | Chi-Square test | **Th**is study was carried out between April 2002 to September 2002 by medical students, which included 857 adults (over 18 years) of either gender with the mean age of 35(14). Study sample had 172 males with mean age 39± 17 year and 685 females with mean age 34± 13 years. 53% of the of the participants were under 35 years of age. Participants were classified as normal, high normal or hypertensives. 223(26%) had HTN with newly diagnosed cases were 130(15%) while 93 (11%) knew their condition. Out of 857 subjects 712(83.08) ever married and 145(16.92) singles participants. | The prevalence of HTN (**BP≥140/90 mm Hg or use of medication**) was 223(26%), male 59 (34.3) more prevalent than females 164 (23.9). singles 145(16.92) had lower prevalence 14 (9.6%) than ever married 209 (29.4%). Prevalence increased over age above 35: 29.8% among age- group 35- 44, 49.5% among 45- 54 and among age- group 55 and above 56.4% prevalence was observed. | NR | NR | Gender, Age- groups, Aware of HTN and Marital Status. | Gender-Male (OR 1.7, CI 1.14- 2.42), Age (above 35 years) (OR 5.6, CI 3.9- 8.1) were significant predictors of HTN. | 6 |
| 60 | Shafi et al, 2017, rural central Punjab Pakistan | Aim: To obtain updated information on the prevalence, awareness, treatment and control of HTN through multiple health screening camps in central Punjab.  Design: This cross- sectional study was carried out on data surveyed at multiple location in rural central Punjab. At multiple camps randomly selected participants were interviewed through a pre- tested questionnaire, their BP readings and anthropometric measurements were obtained. | | Multivariate logistic regression along with adjusted and unadjusted odd ratio for significant predictors. | 13,722 participants with median age = 40 years (IQR, 30–50 years), male = 8366 (61%), married =11,672 (85.1%), college education= 3986 (28.9%) and working= 7378 (53.8%) were included. Among patients with hypertension, 62.3% were aware of having high blood pressure; Among all patients, 9934 had no comorbid conditions like diabetes mellitus, cardiovascular disease, or chronic kidney disease. | Hypertension was defined as a prior history of hypertension and/or medical treatment of hypertension and/or a systolic blood pressure above 140 mmHg or diastolic blood pressure above 90 mmHg at the health screening camp. Crude prevalence of hypertension was 35.1% and age-standardized prevalence was 34.4%. | Among patients with hypertension, 62.3% were aware of having high blood pressure; among these patients, 75.3% were already on treatment for hypertension. | NR | Age, gender, working, family history of HTN, history of chronic kidney disease, history of diabetes, history of cardiovascular disease, family history of cardiovascular disease, alcohol intake, smoking, marital status, educational status and physical activity. | **Variable: Unadjusted OR (95%CI) P-value Adjusted OR (95%CI) P-value**  **Age:** 1.045 (1.041–1.049) <0.001 1.048 (1.043–1.053) <0.001  **Male sex:** 0.62 (0.56–0.68) <0.001 0.71 (0.59–0.84) <0.001  **Working**: 0.58 (0.52–0.64) <0.001 0.83 (0.71–0.98) 0.03  **Family history of HTN**:1.79 (1.62–1.97) <0.001 2.04 (1.80–2.30) <0.001  **History of chronic kidney disease**:1.99 (1.73–2.29) <0.001 1.85 (1.57–2.17) <0.001  **History of diabetes mellitus:** 3.41 (2.98–3.90) <0.001 1.95 (1.68–2.27) <0.001  **History of cardiovascular disease:** 5.2 (3.97–6.82) <0.001 2.98 (2.20–4.0) <0.001  **Body mass index:** 1.08 (1.07–1.09) <0.001, 1.06 (1.05–1.07) <0.001 | **8** |
| 61 | Shah et al, 2001,  District_ Ghizar northern area of Pakistan | Aim: To study the prevalence and determinants of hypertension in rural area Design: Cross- sectional Community based study, using stratified random sampling techniques in 16 villages. | | Logistic Regression, Chi_ Square Test | 4203 adults (age >18 years) living mountainous (rural) area, 2797 (66.5% females (mean age 36 years), men’s mean age 42. | The average of 3 readings of BP was taken by a trained interviewer (manually using mercury Sphygmomanometer). For men, the mean (SD) BP (mm Hg), systolic was 125 (19) and diastolic 80 (12); for women, it was 125 (22) and 78 (14), respectively. Prevalence of HTN **(BP≥140/90 or currently taking antihypertensive medication) =** 626 (15%) | 60 (10%) of the hypertensives (n=626). | NR | Gender, Age, BMI, use of snuff, Salted Tea use, Smoking, Use of wine, family history of hypertension | **Variable: AOR (95% CI)**  Age:  30-39 2.76(1.94,3.92)  40-49  6.40(4.56,8.99)  50-59 12.10(8.55,17.12)  ≥60 13.21(8.78,19.86)  Higher BMI (>25):  2.01(1.51,2.67)  Family history:  1.90 (1.49,2.42) | **8** |
| 62 | Shams et al,2015, Karachi | Aim: To determine the frequency and risk factors of obesity and hypertension among female medical students.  Design: The design of the study was cross- sectional. Data on base line characteristics, followed by BP readings and anthropometric measurements was recorded on a structured questionnaire. Students suffering from diabetes, had any systemic illness or taking corticosteroids were excluded from study population. | | Chi Square Test | This study was carried out between March 2014 to June 2014 on 307 female MBBS and BDS students, excluding Students suffering from diabetes, had other systemic illness or taking corticosteroids. Mean age of the participants was 22± 1.36. Out of 307 participants, 91 were obese, 15 hypertensives and 11(12%) subjects had both obesity and hypertension. Participants with family history of diabetes, hypertension, heart disease and obesity were 135 (44%), 147 (45%), 60 (19.5%) and 58 (19%) respectively. The mean value of sleep hours on weekdays 6.73±1.77(ranging 2- 12 hour), sleeping hour on holiday 9.05± 2.16 (ranged 1-18 hour), mean outdoor games and exercise/ week 2.42± 2.95 (ranged 0- 14) and mean sitting time/ day 3.99± 1.96 (ranging 0- 10). | BP was measured using mercury sphygmomanometer in sitting position on both arms. JNC-VIII guideline was used to define HTN. Prevalence of HTN **(BP≥140/90 or use of medication)** = 15 (4.88%) with mean SBP= 113.91± 9.45 and mean DBP= 74.36±8.49. | NR | 11(12%) subjects had both obesity and hypertension. | Family history, sleeping hours, dietary preferences, screen time, sedentary time and outdoor activity. | Excessive use of meat product, chocolates, soft drinks, over sleep, family history of heart disease were found to be predicting variable (effect sizes not reported, only p-value given). | 5 |
| 63 | Siddique et al, 2005, Karachi | Aims: To asses risk factors for hypertension among adults in squatter settlement of Karachi. Design: Cross- sectional study was undertaken using random sampling techniques to select 64 houses for the survey. A pre-tested questionnaire was used for the data collection | | Chi- Square test and t- test were used for the analysis. Odd Ratios for significant predictors were calculated. | This study comprised a total of 327 participants both male and female aged 15 and above in low socio- economic area Squatter Settlement, Karachi. Individuals with known history of hypertension were excluded from study sample. In remaining 327 165(50.5) Males and 162(49.5) females were recruited. | BP was measured on the right arm in sitting position using calibrated aneroid sphygmomanometer and recorded on questionnaire. JNC-VIII criteria  was used for the diagnoses of HTN. This study demonstrated overall 15% prevalence with 19 (14%) in females and 11 (17.5%) in males. | NR | 19 (9.5%) of 200 were diabetic. | Age, gender, occupation, education, family history of hypertension, personal history of diabetes, smoking and BMI. | Diabetic individuals were significantly more likely (p<0.001) to be hypertensive (Odd Ratio= 9.72, CI= 3.1-33.1).  The mean age of hypertensives was (38.7 + 16.5), was significantly greater as compared to normotensive with the mean age of (29.2 + 13.6). Similarly, significant difference was observed in mean BMI of hypertensives (25.6 + 4.5) verses normotensives (22.9 + 5.0 kg/m2) | 6 |
| 64 | Sikandar et al 2015, Peshawar University Campus. | **Aim:** To find the risk factors of hypertension among teachers of the University of Peshawar.  **Design:** Between June to August 2010, a cross-sectional observational study was conducted in all departments of University of Peshawar including UET, KMC, KCD, Jinnah College for women, Home Economic college, Islamia college, Agricultural University and Institute of management sciences. Data was collected through a pre-tested (via pilot study) close-ended questionnaire followed by physical examination (BP and anthropometric measurements). Participants were selected through simple random sampling technique. | | Descriptive analysis | A total of 500 subjects aged above 25 years, of either gender was investigated after obtaining their verbal consent. Maximum number of subjects 183 (36.6%) were observed in age group 41-50. 353(70.6) males and 147(29.4%) females comprised 38(7.6%) professors, Assistant professor 92(18.4), 79(15.8) Associate professor, 161(32.2) lecturers, demonstrator 108(21.6) and intern 22(4.4). The study had shown 107 (21.4%) overweight subjects and 27 (5.4) had obesity. Among all 500 cases 103 (20.6) had family history of hypertension, 116 (23.2) had family history of diabetes and 89 (17.8) had family heart disease. Most of the participants 418 (83.6) had stress, only 94 (18.8) exercise daily and 133 (26.6) smoker males observed as doing their regular BP checking. | After completing the questionnaire, each participant’s BP was measured using an aneroid sphygmomanometer. Two readings were obtained two minutes apart and the average of these two reading were considered for the analysis. The prevalence of HTN according to JNC_VII guideline was **(BP≥140/90 or use of antihypertensive medication)** 128(25.6%), with 87/353 (27.5%) in males and 41/147 (27.9%) in women. | 56(11.2%) were treated with medication for lowering BP. | NR | Age, gender, marital status, designation, family history of diseases, smoking, diet, life style, exercise, stressed or tensed. | Prevalence was higher 9/14 (64.3%) in the age above 60, those who had family history of HTN had 103 (42.7%) prevalence, among overweight 41(38.3) and obese 15 (55.6) prevalence seen. Prevalence among smokers 39(29.3), stressed 112 (26.8) and those leading active life was 22 (15.4). | **6** |
| 65 | Sughis et al, 2012, Lahore. | Aim: To estimate the relationship between blood pressure and exposure to traffic related air pollution among children. Design: This was a cross- sectional study recruited all willing children (between age 8 and 12 years) from two schools of low and high polluted area of Lahore City. At the study site the maximum and minimum sound level was measured using sound level meter and atmospheric particulate matter (PM) was measured with laser operated device 24 h before examination day. Data on life style followed by anthropometric and clinical measurements including urine test for analysing creatinine and BP readings were recorded on a pre- tested questionnaire originally made for the international study of asthma and allergies in childhood. | | Student t test, Wilcoxon’s test, Chi Square test and Logistic Regression. | This study recruited 166 school children between age 8 and 12 years in schools situated in high and low air polluted areas. Of these 166 subjects 73 (Male 46 (63%), Female 27 (37%)) were from low pollution school and remaining 93 (Male 46 (49.4%), Female 47 (50.5%)) from high pollution school. The average age of children was 9.9 years. In both the schools most of the children had high socioeconomic class (41(57%) vs 39 (42%)). The median value of weight (27 kg), height (134 cm) and BMI (15.3) was same in both the schools. No significance difference was seen in outdoor temperature 15.5 ^0^C (3.3) vs 14.9 ^0^C (4.5) of both schools. Indoor particulate matter (222.9 (119.4) vs 590.7 (219.4)) and humidity (58.4 (16.4) vs 67.1 (18.5)) was higher at high pollution schools. | Participants were classified as normal (systolic <120 mmHg and diastolic <80 mmHg), pre- hypertensive (systolic 120–139 mmHg or  diastolic 80–89 mmHg) and hypertensive (BP**≥140/90 mm Hg**) after measuring their BP in five consecutive reading with initial rest time of five minutes. According to JNC VII hypertension had not been seen in any child whereas 14 (19%) in the low polluted school and 14 (19%) in the high pollution school had pre- hypertension. | NR | NR | Gender, age, BMI, passive smoking, socio- economic status, urinary sodium, potassium and creatinine | High salt intake and exposure to traffic noise and air pollution were significant predictors of high blood pressure among children. | 6 |
| 66 | Tareen et al, 2011, Faisal Abad, Northeast of Punjab. | Aims: To investigate the association of social class (or location of residence) with cardiovascular risk factors (mainly hypertension and diabetes mellitus) in Pakistan.  Design: This analysis is based on the baseline data collected for a cohort Urban Rural Chronic Diseases Study (URCDS). Hence, the study design was cross- sectional. | Logistic Regression Model, Unadjusted and Adjusted analyses were used. | | This study was conducted between January to June 2009 among inhabitant of Faisal Abad including one peripheral rural area of Punjab. Sample size =2495, recruited healthy participants aged 30 to 75 years. All residents of the local community were invited to participate, Male were 1271(50.9%) Female 1224(49.1%), mean age of the participants =50.30 ± 12.49. Among 2495 subjects rural dwellers were 1417(56.8%) and urban 1078(43.3%). Lower social class (manual workers) were 44% whereas middle class-(non-manual workers) were 40%, high social class (professionals) was only 393(15.7). Among total sample size overweight were 1138(45.6), obese 771(30.9) and only 556(22.3) had desirable weight. | The selected participants were required to visit the research project clinics at three different hospitals of Punjab for BP, anthropometric and blood glucose measurements. The overall prevalence of diabetes and HTN **(if diagnosed previously or BP>140/90)** was 16.6% and 24.2% respectively. | NR | NR | Age, Sex, BMI, Social class by occupation, Area of Residence | Unadjusted Odd Ratio for Social Class of Non-Manual Workers (UOR 0.65, CI 0.50-0.83), Manual Workers (UOR 0.50, CI 0.39-0.64) and Urban dwellers (UOR 2.82, CI 2.33-3.41) showed significant association with HTN. After adjustment only Urban dwellers were found associated with hypertension (AOR 3.03, CI 2.41-3.82) and Diabetes (AOR1.77, CI 1.37-2.29). | 8 |

**^a^** Study are added in the alphabetical order of the 1^st^ author name and reference number.

**^b^** In some of the studies, the analysis assessing the association is based on a sub-sample of the study due to missing data, in the results section of the main paper the total number of participant (99,391) exclude those participants whose data was not included in the association analysis.

**^c^** NOQAS= Newcastle Ottawa Quality Assessment Scale

**NR:** Not Reported (Not undertaken)
