# Supplementary material for: Factors associated with hypertension in Pakistan: A systematic review and meta-analysis
Source: PLoS One. 2021 Jan 29;16(1):e0246085. doi: 10.1371/journal.pone.0246085 (PMC7845984; doi:10.1371/journal.pone.0246085)
Supplement: S2 Table — (DOCX) [file pone.0246085.s030.docx]

**S2** **Table : Assessing Quality of the Cross-Sectional Studies using Modified Newcastle Ottawa Quality Assessment Scale (MNOQAS).**

| **Study ref. no** | **Study** | **Selection** | | | | **Comparability** | | **Outcome** | | **MNOQAS** |
| --- | --- | --- | --- | --- | --- | --- | --- | --- | --- | --- |
|  |  | **R** | **S** | **NR** | **AE** | **D** | **Ana.** | **Assess.** | **SA** | Score |
| 30 | Akatsu et al, 1996, Karachi | * | * | * | * |  |  | * | * | 6 |
| 31 | Aslam et al, 2013, Karachi |  | * | * | * |  |  | * | * | 5 |
| 32 | Aziz et al, 2005, Karachi | * | * | * | * |  |  | * | * | 6 |
| 33 | Bilal et al, 2019, Multan | * | * | * | * |  |  | * | * | 6 |
| 34 | Farooq et al, September 2016, Lahore Pakistan. |  | * | * | * |  |  | * |  | 4 |
| 35 | Gul et al, 2015, Hayatabad Peshawar |  | * | * | * |  |  | * | * | 5 |
| 36 | Gupta et al, 2017, Karachi | * | * | * | * |  | * | * | ** | 8 |
| 37 | Habib et al, 2019, district Mianwali, Punjab | * | * | * | * |  |  | * | * | 6 |
| 38 | Humayun et al, 2009, Peshawar |  | * | * | * |  |  | * | * | 5 |
| 39 | Ikramullah et al, 2014, Peshawar | * | * | * | * |  |  | * | * | 6 |
| 40 | Ilyas et al, 1980, Peshawar |  | * | * | * |  |  | * | * | 5 |
| 41 | Ishtiaq et al, 2017, Rawalpindi Islamabad, Pakistan | * | * | * | * |  |  | * | * | 6 |
| 42 | Jafar et al, 2003, rural and urban areas of all four provinces of Pakistan. | * | * | * | * |  | * | * | ** | 8 |
| 43 | Jawed et al, 2017, Faisalabad | * | * | * | * |  | * | * | ** | 8 |
| 44 | Khan et al, 2012, Peshawar | * | * | * | * |  |  | * | * | 6 |
| 45 | Khan et al, 2015, Khyber Pakhtunkhwa | * | * | * | * |  |  | * | * | 6 |
| 47 | Manzoor et al, 2019, district Faisalabad | * | * | * | * |  |  | * | * | 6 |
| 53 | Nawaz et al, 2010, Lahore Pakistan. |  | * | * | * |  | * | * | ** | 7 |
| 54 | Rafique et al, 2002, Agha Khan University Karachi. | * | * | * | * |  |  | * | * | 6 |
| 56 | Rahman et al, 2013, Karachi. |  | * | * | * |  | * | * | * | 6 |
| 57 | Raza et al, 2019, Karachi. | * | * | * | * |  |  | * | * | 6 |
| 58 | Rehman et al, July 2016, Peshawar | * | * | * | * |  |  |  | * | 5 |
| 59 | Safdar et al, 2004, Karachi | * | * | * | * |  |  | * | * | 6 |
| 60 | Shafi et al, 2017, rural central Punjab Pakistan | * | * | * | * |  | * | * | ** | 8 |
| 61 | Shah et al, 2001,  District_ Ghizar northern area of Pakistan | * | * | * | * |  | * | * | ** | 8 |
| 62 | Shams et al,2015, Karachi |  | * | * | * |  |  | * | * | 5 |
| 63 | Siddique et al, 2005, Karachi | * | * | * | * |  |  | * | * | 6 |
| 64 | Sikandar et al 2015, Peshawar University Campus. | * | * | * | * |  |  | * | * | 6 |
| 65 | Sughis et al, 2012, Lahore | * | * | * | * |  |  | * | * | 6 |
| 66 | Tareen et al, 2011, Faisal Abad, Northeast of Punjab. | * | * | * | * |  | * | * | ** | 8 |

**R:** Representativeness of the population (i.e., Is the sample ‘truly’ or ‘somewhat’ representative of the community (population studied).

**S:** Sample size (i.e., is the sample size justified and sufficient?)

**NR:** Non-respondents: Satisfactory response rate was achieved, and respondents and non-respondents were comparable with regards to participants’ characteristics.

**AE:** Exposure was ascertained based on secure medical records or structured interview)

**D:** Study design controlled for the important factor such as socio-economic status.

**Ana:** The statistical analyses adjust results for additional potential confounders such as age, sex, smoking, medication other than hypertensive).

**Assess:** If the outcome “hypertension” is self-reported, is it validated by the BP measure or medical records)

**SA:** Is statistical analysis clearly described* and is it appropriate**?
