# Supplementary material for: Factors associated with hypertension in Pakistan: A systematic review and meta-analysis
Source: PLoS One. 2021 Jan 29;16(1):e0246085. doi: 10.1371/journal.pone.0246085 (PMC7845984; doi:10.1371/journal.pone.0246085)
Supplement: S3 Table — (DOCX) [file pone.0246085.s031.docx]

**S**3 **Table : Assessing Quality using Newcastle Ottawa Assessment Scale (NOQAS) for Case- Control Studies.**

| **ref. no** | **Study** | **Selection** | | | | **Comparability** | | **Exposure** | | | **Score** |
| --- | --- | --- | --- | --- | --- | --- | --- | --- | --- | --- | --- |
|  |  | **ACD** | **ROC** | **SOC** | **DOC** | **D** | **Ana.** | **AE** | **SMA** | **SNR** | **NOSS** |
| 46 | Malik et al, 2013, Lahore | * |  | * | * |  |  | * | * | * | 6 |
| 48 | Mubarik et al, 2019, Rawalpindi | * |  | * | * |  | * | * | * | * | 7 |
| 49 | Mushtaq et al, 2014 | * |  | * | * | * |  | * | * | * | 7 |
| 50 | Mushtaq et al, 2014, Lahore | * |  | * | * | * | * | * | * | * | 8 |
| 51 | Mushtaq et al, 2014, Lahore Punjab | * | * | * | * | * |  | * | * | * | 8 |
| 52 | Mushtaq et al, 2015, Lahore | * |  | * | * | * |  | * | * | * | 7 |
| 55 | Rafique et al, 2019, Islamabad | * |  | * | * | * | * | * | * | * | 8 |

ACD: Adequate case diagnoses (With standard criteria)

**ROC**: Representativeness of the cases (consecutive or obviously representative series of cases)

**SOC**: Selection of controls (Community controls, i.e. same community as cases and would be cases if had outcome)

**DOC**: Definition of controls (No history of disease)

**D**: Study design controls for most important factor.

**Ana:** The statistical analyses adjust results for additional potential confounders

**AE:** Exposure was ascertained based on secure medical records or structured interview

**SMA: S**ame method of ascertainment for cases and controls.

**SNR:** Same Non- response rate for both the groups

**ST:** Statistical test (i.e., the statistical test used for the analysis is clearly described and appropriate?
