# Supplementary material for: Factors associated with hypertension in Pakistan: A systematic review and meta-analysis
Source: PLoS One. 2021 Jan 29;16(1):e0246085. doi: 10.1371/journal.pone.0246085 (PMC7845984; doi:10.1371/journal.pone.0246085)
Supplement: S4 Table — (DOCX) [file pone.0246085.s032.docx]

**S4 Table: PRISMA 2009 Checklist**

| **Section/topic** | | **#** | | **Checklist item** | Reported on page # |
| --- | --- | --- | --- | --- | --- |
| **TITLE** | | | | |  |
| Title | | 1 | | Identify the report as a systematic review, meta-analysis, or **both.** | Title Page before 1 |
| **ABSTRACT** | | | | |  |
| Structured summary | | 2 | | Provide a structured summary including, as applicable: background; objectives; data sources; study eligibility criteria, participants, and interventions; study appraisal and synthesis methods; results; limitations; conclusions and implications of key findings; systematic review registration number. | 1-2 |
| **INTRODUCTION** | | | | |  |
| Rationale | | 3 | | Describe the rationale for the review in the context of what is already known. | 2-4 |
| Objectives | | 4 | | Provide an explicit statement of questions being addressed with reference to participants, interventions, comparisons, outcomes, and study design (PICOS). | 3-4 |
| **METHODS** | | | | |  |
| Protocol and registration | | 5 | | Indicate if a review protocol exists, if and where it can be accessed (e.g., Web address), and, if available, provide registration information including registration number. | NA |
| Eligibility criteria | | 6 | | Specify study characteristics (e.g., PICOS, length of follow-up) and report characteristics (e.g., years considered, language, publication status) used as criteria for eligibility, giving rationale. | 5-6 |
| Information sources | | 7 | | Describe all information sources (e.g., databases with dates of coverage, contact with study authors to identify additional studies) in the search and date last searched. | 5-6, 9-10 |
| Search | | 8 | | Present full electronic search strategy for at least one database, including any limits used, such that it could be repeated. | 5, 9, (S5Table) |
| Study selection | | 9 | | State the process for selecting studies (i.e., screening, eligibility, included in systematic review, and, if applicable, included in the meta-analysis). | 5-6, 9 |
| Data collection process | | 10 | | Describe method of data extraction from reports (e.g., piloted forms, independently, in duplicate) and any processes for obtaining and confirming data from investigators. | 6. |
| Data items | | 11 | | List and define all variables for which data were sought (e.g., PICOS, funding sources) and any assumptions and simplifications made. | 6 (S1Table 1) |
| Risk of bias in individual studies | | 12 | | Describe methods used for assessing risk of bias of individual studies (including specification of whether this was done at the study or outcome level), and how this information is to be used in any data synthesis. | 7, 11  , & (S2, S3 Tables ) |
| Summary measures | | 13 | | State the principal summary measures (e.g., risk ratio, difference in means). | 7-8, 12-15, 33-36 |
| Synthesis of results | | 14 | | Describe the methods of handling data and combining results of studies, if done, including measures of consistency (e.g., I^2^) for each meta-analysis. | 7-8, 11-15, Table 1 (Sup. Figures 1-28) |
| Risk of bias across studies | 15 | | Specify any assessment of risk of bias that may affect the cumulative evidence (e.g., publication bias, selective reporting within studies). | | 7, NOQAS, p.15, (S1-3-Tables S1-28 Figures) |
| Additional analyses | 16 | | Describe methods of additional analyses (e.g., sensitivity or subgroup analyses, meta-regression), if done, indicating which were pre-specified. | | 8-9, 14-16 |
| **RESULTS** | | | | |  |
| Study selection | 17 | | Give numbers of studies screened, assessed for eligibility, and included in the review, with reasons for exclusions at each stage, ideally with a flow diagram. | | 9-10 |
| Study characteristics | 18 | | For each study, present characteristics for which data were extracted (e.g., study size, PICOS, follow-up period) and provide the citations. | | 9-10, (S1 Table) |
| Risk of bias within studies | 19 | | Present data on risk of bias of each study and, if available, any outcome level assessment (see item 12). | | 11, 15, NOQAS (S1-3 Tables, S1-27 Figs) |
| Results of individual studies | 20 | | For all outcomes considered (benefits or harms), present, for each study: (a) simple summary data for each intervention group **(b)** effect estimates and confidence intervals, ideally with a forest plot. | | 11-15, (Table 1, Figs 2, 3, (S1 Table , S1-28Figs) |
| Synthesis of results | 21 | | Present results of each meta-analysis done, including confidence intervals and measures of consistency. | | 12-15, (Table 1, Figs 2, 3)  S1-28 Figs) |
| Risk of bias across studies | 22 | | Present results of any assessment of risk of bias across studies (see Item 15). | | 11, 15, 19-20. (NOQAS, S2-3 Tables) |
| Additional analysis | 23 | | Give results of additional analyses, if done (e.g., sensitivity or subgroup analyses, meta-regression [see Item 16]). | | 12-16, (Table 1, Figs 2-3) (S1-28 Figs). |
| **DISCUSSION** | | | | |  |
| Summary of evidence | 24 | | Summarize the main findings including the strength of evidence for each main outcome; consider their relevance to key groups (e.g., healthcare providers, users, and policy makers). | | 17-19. |
| Limitations | 25 | | Discuss limitations at study and outcome level (e.g., risk of bias), and at review-level (e.g., incomplete retrieval of identified research, reporting bias). | | 19-20 |
| Conclusions | 26 | | Provide a general interpretation of the results in the context of other evidence, and implications for future research. | | 17-18, 21-22 |
| **FUNDING** | | | | |  |
| Funding | 27 | | Describe sources of funding for the systematic review and other support (e.g., supply of data); role of funders for the systematic review. | | NA |

From: Moher D, Liberati A, Tetzlaff J, Altman DG, The PRISMA Group (2009). Preferred Reporting Items for Systematic Reviews and Meta-Analyses: The PRISMA Statement. PLoS Med 6(7): e1000097. doi:10.1371/journal.pmed1000097.

For more information, visit: www.prisma-statement.org.

NOQAS= Newcastle Ottawa Quality Assessment Scale.

Sup.= Supplementary.

#: Data extraction from published manuscript only.

Tables S1-S3= Tables 1-3 provided in this document.

NA=Not Applicable (Not undertaken)

The number in the last column refers to the page number of the manuscript without track changes or this supplementary document if specified.

**Supplementary figures:**

**Supplementary** Figure 1 Funnel plots assessing publication bias in the results for age-groups

1_1 Age group: 30-39


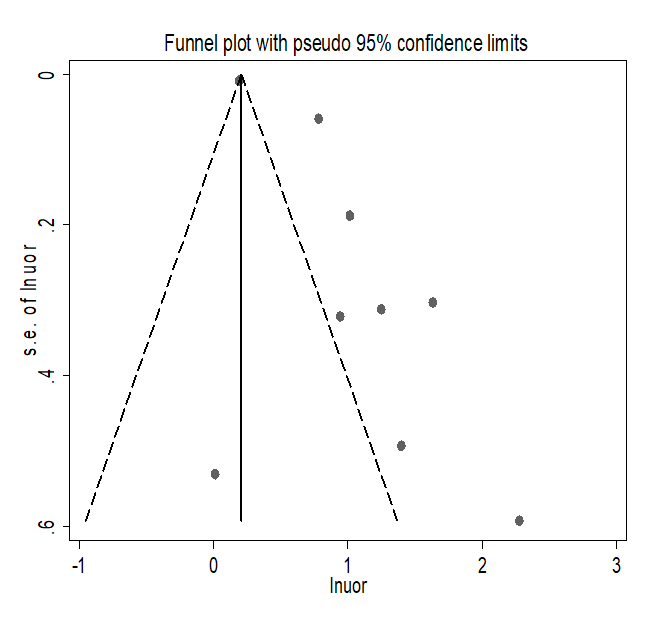


1_2 **Age group: 40-49**

**
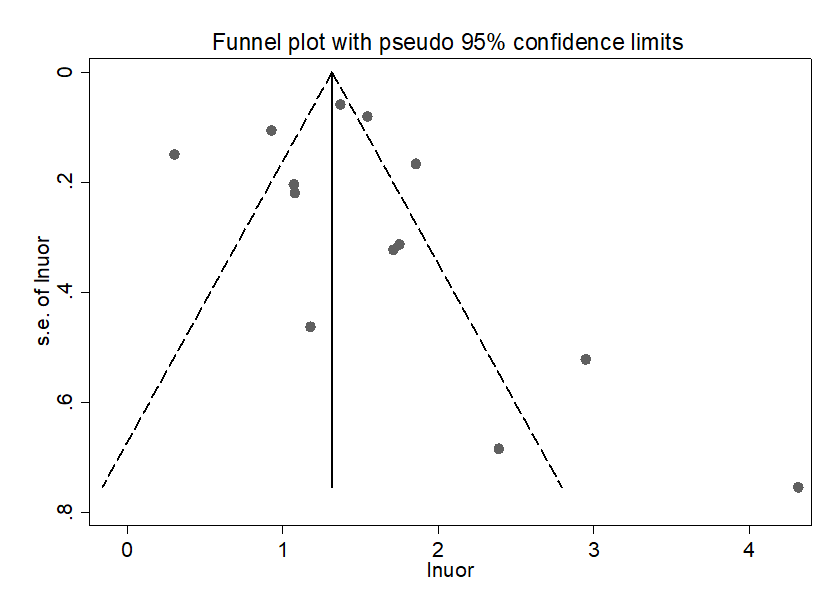
**

1_3 Age group: 50-59

1_4 Age group≥60

**Supplementary** **Figure 2 Forest plot from the Meta-analysis (using Random Effect Model) of gender**

**Supplementary** Figure 3 Funnel plots assessing publication bias in the results for gender

**Supplementary Figure 4 Forest plot from the Meta-analysis of marital status**

**Supplementary** Figure 5 Funnel plots assessing publication bias in the results for marital status

**Supplementary Figure 6 Forest plot from the Meta-analysis of the levels of education**

**Supplementary Figure 6 Funnel plots assessing publication bias in the results for the level of education**

**Supplementary Figure 7 Forest plot from the Meta-analysis of income status**

**Supplementary** Figure 8 Funnel plots assessing publication bias in the results for income status

**Supplementary Figure 9 Forest plot from the Meta-analysis of physical activity (active versus sedentary)**

**Supplementary** Figure 10 Funnel plots assessing publication bias in the results for physical activity

**Supplementary Figure 11 Forest plot from the Meta-analysis of unrestricted salt use**

**Supplementary** Figure 12 Funnel plots assessing publication bias in the results for unrestricted use of salt in the food

**Supplementary Figure 13 Forest plot from the Meta-analysis of tobacco use**

**Supplementary** Figure 14 Funnel plots assessing publication bias in the results for tobacco use

**Supplementary Figure 15 Forest plot from the Meta-analysis of family history of hypertension**

**Supplementary** Figure 16 Funnel plots assessing publication bias in the results for family history of hypertension

**Supplementary Figure 17 Forest plot from the Meta-analysis body mass index(BMI) groups**

**Supplementary Figure 18 Funnel plots assessing publication bias in the results for BMI (overweight and obese)**

**18_1 overweight**

**18_2 Obese**

**Supplementary Figure 19 Forest plot from the Meta-analysis having diabetes**

**Supplementary Figure 20 Funnel plots assessing publication bias in the results for having diabetes**

**Supplementary Figure 21 Forest plot from the Meta-analysis having anxiety**

**Supplementary Figure 22 Funnel plots assessing publication bias in the results for having anxiety**

**Supplementary Figure 23 Forest plot from the Meta-analysis having stress**

**Supplementary** Figure 24 Funnel plots assessing publication bias in the results for having stress

**Supplementary Figure 25 Forest plot from the Meta-analysis for anger-in (& -control)**

**Supplementary Figure 26 Funnel plots assessing publication bias in the results for anger-in (& -control)**

**26_1: anger-in**

26_2 anger-control

**Sensitivity Analyses:**

**Supplementary Figure 27 Forest plots for the meta-analyses of factors with effect estimates from three or more available high-quality studies (NOQAS sore ≥ 7).**

Supplementary Table 5: Search Syntaxes

| No | Search Syntax |  |
| --- | --- | --- |
| 1 | (Predictor OR factors OR determinant OR characteristics OR component) AND (psychological OR socioeconomic OR sociodemographic OR clinic$ OR behavior$ OR sibling OR family$ OR history) AND hypertension AND Pakistan |  |
| 2 | (Predictors OR factors OR determinant OR characteristics OR component) AND (psychological OR socio$ OR clinic$ OR behavior$ OR sibling OR family$ OR history OR diabetes OR cardiovascular OR heart OR liver) AND hypertension AND Pakistan |  |
| **In addition, we ran the following reduced searches to make sure that we have not missed study:** | | |
| 3 | Predictors AND hypertension AND Pakistan |  |
| 4 | (Factors OR Association) AND hypertension AND Pakistan |  |
| 5 | characteristics AND hypertension AND Pakistan |  |
| 6 | component AND hypertension AND Pakistan |  |
