# Supplementary material for: Factors associated with hypertension in Pakistan: A systematic review and meta-analysis
Source: PLoS One. 2021 Jan 29;16(1):e0246085. doi: 10.1371/journal.pone.0246085 (PMC7845984; doi:10.1371/journal.pone.0246085)
Supplement: S5 Table — (DOCX) [file pone.0246085.s033.docx]

**S5 Table : Electronic Search Syntaxes**

| **No** | **Search Syntax** |  |
| --- | --- | --- |
| 1 | (Predictor OR factors OR determinant OR characteristics OR component) AND (psychological OR socioeconomic OR sociodemographic OR clinic$ OR behavior$ OR sibling OR family$ OR history) AND hypertension AND Pakistan |  |
| 2 | (Predictors OR factors OR determinant OR characteristics OR component) AND (psychological OR socio$ OR clinic$ OR behavior$ OR sibling OR family$ OR history OR diabetes OR cardiovascular OR heart OR liver) AND hypertension AND Pakistan |  |
| **In addition, we ran the following reduced searches to make sure that we have not missed study:** | | |
| 3 | Predictors AND hypertension AND Pakistan |  |
| 4 | (Factors OR Association) AND hypertension AND Pakistan |  |
| 5 | characteristics AND hypertension AND Pakistan |  |
| 6 | component AND hypertension AND Pakistan |  |
