# Supplementary material for: Factors associated with hypertension in Pakistan: A systematic review and meta-analysis
Source: PLoS One. 2021 Jan 29;16(1):e0246085. doi: 10.1371/journal.pone.0246085 (PMC7845984; doi:10.1371/journal.pone.0246085)
Supplement: Attachment — Submitted filename: MR-Replies to revierwers comments090920.docx [file pone.0246085.s034.docx]

**Our replies to the Reviewers’ Comments:**

**We thank the reviewers for their valued comments. In our replies below, the page numbers refer to the pages of the manuscript copy with track changes.**

**Reviewer #1:** This is a valuable project however it has lots of grammatical mistakes which made it difficult to read. manuscript is very long and in all parts it needs to be summarized. In this format of reporting I could not recommend it for publication.

**Reply:  Thank you, we agree that our writing may lack grammatical accuracy. We have made the changes, please see them in the copy with track changes. For further grammatical corrections and editing, we will use the services of a professional editor “Editage” if the manuscript is accepted for publication. We believe this will remove the English language flaws.**

**Furthermore, this is a comprehensive systematic review and meta-analysis; therefore, the size of the manuscript is larger than the normal manuscript (20 pages excluding tales, figures, and references).**

**Reviewer #2:** Riaz and colleagues conducted a systematic review and meta-analysis to find out the factors associated with hypertension in Pakistan. The topic is unique, however I have several comments/concerns.

Introduction:
1. In the first paragraph the authors mentioned about the definition of hypertension by several guideline. However, they did not mention about the 2017 American College of Cardiology/American Heart Association Hypertension Guideline. That should be mentioned.

**Reply: Thank you for pointing this out. The aim of the paragraph was to highlight the importance of understanding the variability of guidelines among different organizations. Now, we have added on the 2017 American College of Cardiology/American Heart Association Hypertension Guideline to the paragraph (page 3, lines 50-59, track changes copy).**

2. Line 67: In which year that rural survey was conducted? please mention the year.

**Reply: Thank you for pointing this out, the year is 2001, we have added this to the text on page 5 line 97.**

3. Line 71-72: “In Pakistan, 12% of all deaths are attributed to circulatory diseases annually [4].” This sentence is inconsistent with this paragraph and should be removed.

**Reply:   Thank you, we have deleted this.**

4. The introduction lacks the knowledge gap on this topic. Please state the knowledge gap. The risk factors are already known as stated in the introduction, then what is the purpose of conducting this systematic review and meta-analysis? This should be mentioned.

**Reply:  We respect the reviewer comments on this. However, we would like to mention that in the last paragraph of introduction, it is stated that** **“*A wide range of studies have been conducted on factors associated with hypertension in Pakistan, but the findings of an individual study cannot establish any clinical decision. In this regard systematic reviews are highly valued as they critically appraise all the available evidence, combine the results and reach a better conclusion about a research topic [24]. In this study, we report a thorough systematic review and meta-analysis of the literature to make valid inferences, give greater consistency and internal validity of the results. Also, we highlighted the poor quality of existing literature on hypertension and related methodological issues. This review will help in developing useful public health and clinical strategies establishing to control hypertension and decrease the burden of morbidities and mortality associated with hypertension in Pakistan*”. We must mention here that we have now made further changes to this paragraph to make this clearer (page 7-8, lines 125-140.**

Methods:
5. The authors mentioned about the time period in which they searched the literature. However, they did not mention the time period between which the articles were selected. For example between 14th August 1947 (the day Pakistan became a sovereign nation) till 31st December 2019. This must be mentioned.

**Reply:  Line 256-258 in the results section, we stated “These studies were conducted in various parts of the country covering the whole geographic area of Pakistan and were published between 1980 and 2019”. We did not impose any restrictions on time period in the search.**

6. What are the key words they used to search the literature? Please mention that in details for each dataset.

**Reply: A set of keywords is already provided in section “Search strategy” on page 7-8, line 155-160. The same set of keywords has been used for the search in each database. Based on these terms, we created computer syntaxes for online searches in the databases (please see Supplementary Table 5, supplement document page 85).**

7. The authors included studies which objectively measured Blood Pressure as well as the study which included self reported information of hypertension. Were both type of studies included in the meta analysis? If yes, how the authors dealt with the potential measurement error and misclassification bias?

**Reply: Out of 37 included studies, there was only one study which reported self-reported information of hypertension. However, this study considered the use BP lowering medication instead of the standard diagnostic tests. Furthermore, 7 studies were case control with confirmed cases of hypertension. In the remaining 30 cross-sectional studies, one study (Rehman et al, July 2016) reported a self-reported method. Misclassification bias was not suspected with this single study as the use of blood pressure lowering medication was considered with self-reported hypertension.**

8. Why case control and cross sectional stuides were pooled together rather than separate analysis?

**Reply: As we mentioned that we conducted meta-analysis for factors with effect estimates available from three or more studies. For majority of the factors, meta-analysis based on sub-groups is not possible due to the limited number of studies available in each group.**

**In this study, there are only 7 case-control studies out of 37. Majority of them reported a few specific predictors for which meta-analyses are not conducted due to limited number of available studies.**

9. Line 124: What is an old study? Please mention the year.

**Reply: The studies we found in full text were published between year 1980 and 2019. Old studies refer to those articles that had been published before 1980 and an electronic copy of the paper is not available even after a request (via email) was made to the corresponding journal. To make this clear, changes are made in Lines 241-242 and 256-258 (track changes copy).**

Results:
10. Its better to include the study characteristics table in the main text. In the supplementary file, the authors can include the details. However, in the main text succinate information can be given in the table. Please follow Table 1 of this article:
<https://www.ncbi.nlm.nih.gov/pmc/articles/PMC6199753/>
11. It is also better to include the figures of the results of the meta analysis in the main text for the feasibility of the audiences. The figures related to publication bias can be included in the supplementary file.

**Reply: We thank the reviewer for this. However, it is better to keep the table of studies’ characteristics and forest plots in a supplementary file as it retains a lot of information and it can not be presented in the main manuscript. We have presented this kind of work in similar way in our previous systematic review and meta-analysis, please see the article at:** <https://pubmed.ncbi.nlm.nih.gov/29235189/>

**Article, which the reviewer has mentioned above, is based on a single outcome (prevalence of diabetes) and include smaller number of studies, whereas our presentation is for a large number of factors extracted from 37 studies.**

12. In the meta analysis did the authors pooled high quality and poor quality studies together? If yes, they should be separately pooled.

**Reply: It is a good point for a meta-analysis where an investigation of only one outcome/factor or a few factors is involved, and enough number of high-quality studies are available. In this review majority, 24 out of 37 (65%), of the included studies were of poor quality and sub-setting or excluding low quality studies will lose the purpose of this review and meta-analysis. We have discussed these issues in the discussion section. However, we have considered this suggestion for factors where estimates from at least three high quality studies are available. As sensitivity analyses, we have now conducted meta-analyses including only highly quality studies and added the results to Table 1. Please see (pages 10-11, lines #, 232-234; page 14, line # 320-322, tale 1 page 36-39 and supplementary figure 27)**

Discussion:
13. Line 408-412: “This might be because the quality of most of the studies was poor, and assessment (analysis) for these variables may not have been done appropriately. Another reason might be that most of the studies are cross sectional, and those who were hypertensive and used salt, their hypertension might have been controlled through medications.”
It seems the authors pooled the high and poor quality studies together. Also cross sectional and case control studies together. They should have been separately pooled to prevent this dilution.

**Reply: Please see our reply to comments 12 and 8.**

14. Line 420-427: This section is redundant and should be removed.

**Reply: This section highlighted the strength of this study, but as suggested by the reviewer, we have made the changes by removing majority of the section and keep it in brief, please page 20-21 line # 469-478.**

15. The discussion deals with the methodological issues. However, the public health and clinical importance of these findings should be discussed. Otherwise the purpose of this manuscript will be incomplete.16. Please add recommendations for the public health promotion programs of Pakistan.

**I appreciate the comments and agree that it is important to add recommendations for the public health promotion in Pakistan. I would like to draw the reviewers’ attention to a paragraph from line # 420 to line # 442 in the manuscript with track changes. In that section, we highlighted public heath implication. In addition, we now added a new paragraph from line # 531 to line 541 that recommends the general public health priorities in Pakistan in terms research and clinical practices.**

**Reviewer #3:**

1. In this article, Authors had conducted a systematic review and meta-analysis for assessing the factors associated with hypertension in Pakistan. I appreciate their efforts to synthesize the evidence that would be applicable to prevention and management of hypertension in Pakistan. Despite their hard work, there are several gaps in the manuscript that compromise the quality of the study. I would like to suggest the authors to revise the manuscript to address the comments given below and re-submit it.
Introduction: Introduction section is not concise and clear. Citations are missing. Authors need to provide adequate rationale for the study. Would be better if Authors completely revise the introduction section.
**Reply: We thank the reviewer for acknowledging the hard work we have put towards the completion of this research work. As suggested, we have revised the manuscript, in particular, the introduction section. Please see the track changes on pages 3-6.**
Methods
2. Search strategy: How author search PubMed, Elsevier? Search key words are vague. Authors should provide search syntax in supplementary file.

**Reply:  The literature on the topic was searched out in all mentioned databases using the same set of key terms. A list of keywords is already provided in the search strategy (page 7, lines # 155-160). As suggested, we have now provided various computer search syntax in the supplementary document on page 85.**

3. Selection criteria: Did authors use publication/study date as an inclusion criterion?

**Reply:  Sstudies were selected regardless of any consideration of publication date.  All studies conducted in Pakistan reporting factors associated with hypertension (self-reported or clinically diagnosed), and/or reporting hypertension as the analysis outcome were included (Line 164-167). However, we had to exclude those old studies (published before 1980), for which an electronic copy was not available from the journal. We have already mentioned that “were published between 1980 and 2019” in line # 256-258.**

 4. If we are synthesizing the findings of cross-sectional study, should we worry about sample size, sampling methods and other methodological part? For example, did author exclude the study that applied purposive sampling?

**Reply: We have used the Newcastle Quality Assessment Scale for assessing quality of the included studies that consider all these factors, please see tables 2 and 3 in supplementary file, and pages 7 and 11 of the main manuscript. Furthermore, as stated above we have now conducted separate meta-analysis for factors reported by≥ 3 high quality studies. Please see (pages 10-11, lines #, 232-234; page 14, line # 320-322, tale 1: page 36-39 and supplementary figure 27)**

5. What is “old studies”? How did you define hypertension and each associated factor?

**Reply:  Please see replies to comment 9 of reviewer#2 and comment 3 of reviewer#3.**

**They were studies published before 1980, please see the detail in our replies above.**

6. Screening: Did authors use any screening tool (like Endnote)? How the records were imported and screened?

**Reply: We maintained the records manually, and studies were screened by 3 authors independently, according to a pre-defined method (please see the details in “study selection”, Line 128-133).**

7. Data extraction: How did author extracted prevalence estimate from case control study? Were they un/weighted (prevalence) and un/adjusted odds ratio? Which one did you pick up?

**Reply: The detail about effect estimates (Odds Ratios OR) is provided in the meta-analysis section, line 162-184. If a study reported an adjusted OR (only a few), we used the adjusted OR in the meta-analysis otherwise we used unadjusted ORs. If a study reported prevalence/proportions in a table or text but did not report unadjusted OR, we extracted the prevalances and computed the unadjusted OR for meta-analyses.**

8. Quality assessment: What is the purpose of categorizing low- or high-quality study? Did you have sensitivity analysis based on that?

**Reply: The main purpose of “categorizing low- or high-quality study” to describe the quality of the studies and see what proportion of good quality studies exists among the included studies. For some of the factors, we will consider the sensitivity analysis in the revised version. Also, please see our reply to comment 12 of reviewer#2. A sensitivity analyses is now added. Please see (pages 10-11, lines # 232-234; page 14, line # 320-322, tale 1 page 36-39 and supplementary figure 27)**

9. Data analysis: It is not clear whether author used the adjusted or unadjusted or both odds ratio in analysis. Not sure how precise would be the estimate if we combine the study having sample size 120 and 43943 or age having 5 years to 80 years or 3% of prevalence with 77% prevalence. How the authors dealt with the study having only female participants or adjusted the survey year?

**Reply: If a study reported an adjusted OR, we used the adjusted OR in the meta-analysis otherwise we used unadjusted ORs. We appreciate the author’s concerns and understand that these factors add heterogeneity among the studies, which is obvious from I^2^ values reported for each pooled result. We know that meta-analysis is not recommended in the presence of high heterogeneity, but the meta-analysis results are still useful to report in this review with a careful implication of the results. It is not easy to conduct meta-regression and report it for a large number of factors while majority of the studies have poor quality. However, we have now added the sensitivity analyses based on high-quality studies, please see our replies above and (pages 10-11, lines #, 232-234; page 14, line # 320-322, tale 1 page 33-37) of the manuscript and supplementary figure 27.**

10. Results: Once authors address the issues raised in methodology sections, then they might come up with additional subgroup analysis/meta-regression analysis or sensitivity analysis. It would be better if authors cite and supply the supplementary file separately. I think Authors should avoid using “predictor” for the factor associated with outcome variables in cross-sectional study.

**Reply: Thanks to the reviewer, we have now added the sensitivity analyses. Please see our replies above and (pages 10-11, lines #, 232-234; page 14, line # 320-322, tale 1 page 33-37) of the manuscript and supplementary figure 27. We have avoided using** “predictor” unless it was required in few places.

**.**

11. Discussion: Compared to other parts, discussion section had better structural presentation. Author should avoid the statement like – line number 380 “Similarly, patients with mental health problems are also at higher risk and require special attention to be prevented from hypertension.”

**Reply: Thank you, we changed the “patients with mental health problems” to patients having psychological problems. Please see line #430.**

12. Conclusion: not available

**Reply: Conclusion is added in the discussion section. We prefer it in this way for this kind of systematic review. However, if required, we can change this and add it under the sub-heading of conclusion.**
