# Supplementary material for: Factors associated with hypertension in Pakistan: A systematic review and meta-analysis
Source: PLoS One. 2021 Jan 29;16(1):e0246085. doi: 10.1371/journal.pone.0246085 (PMC7845984; doi:10.1371/journal.pone.0246085)
Supplement: Attachment — Submitted filename: Response to Reviewers.docx [file pone.0246085.s035.docx]

**Response to Reviewers**

**Manuscript ID:** PONE-D-20-28653
**Manuscript title:** Associated with Hypertension in Pakistan: A Systematic Review and Meta-Analysis

Dear Editor,

We thank PLOS ONE for providing an opportunity to address the reviewers’ comments, revise and resubmit our manuscript. We thank you and the reviewers for taking time to review our manuscript and suggest the changes required. As recommended by PLOS ONE, we have used the services of Editage (www.editage.com) for English language editing. This has improved the flow and the English language of the manuscript considerably.

In addition to this response letter to yours comments, we upload the following: (i) a revised manuscript with track changes, (ii) a clean version of our revised manuscript without tracked changes, (iii) a cover letter to the editor, (iv) a certificate issued by the Editage for English language editing, (v) three word files for figures 1-3 to be added to the main manuscript, and (vi) six supplementary word files as supporting information (in a zipped folder).

We look forward to hearing from you if there is anything else which could improve the manuscript.

Kind Regards

Muhammad Riaz & co-authors

**Comments and Responses**

**Comment 1**

1. Please ensure that your manuscript meets PLOS ONE's style requirements, including those for file naming. The PLOS ONE style templates can be found at

<https://journals.plos.org/plosone/s/file?id=wjVg/PLOSOne_formatting_sample_main_body.pdf> and

<https://journals.plos.org/plosone/s/file?id=ba62/PLOSOne_formatting_sample_title_authors_affiliations.pdf>

**Reply 1**

**Thank you, we have made the changes and our updated manuscript is according to the PLOS ONE's style templates.**

**Comment 2**

2. Please include captions for your Supporting Information files at the end of your manuscript, and update any in-text citations to match accordingly. Please see our Supporting Information guidelines for more information: http://journals.plos.org/plosone/s/supporting-information.

**Reply 2**

**Thank you, we have added captions and short legends for the supporting information under the heading “Supporting Information” after the references. In addition, we now added the supporting information in 6 word files (supplementary).**

**Comment 3**

3. Please upload the completed PRISMA checklist as a separate file.

**Reply 3**

**We have uploaded PRISMA checklist in a separate file as a supporting information.**

Additional Editor Comments

**Comment 4**

I am persuaded by the authors that this extensive amount of work deserves to be published and it is in keeping with the principals and mission of PLoSONE. There are still problems with English and the manuscript needs to be sent out for copy-ediiting for English.

**Reply 4**

**We thank the editor for valued comments, we agree that our manuscript required copy-editing for English language. We have used the services of Editage (www.editage.com) for English language editing. Their certificate is upload in a separate file.**

**Comment 5**

Otherwise since the abstract is going to be what is most likely to be read it should be revised in the following way. Please divide the factors into those positively associated with hypertension and those that are negatively associated. The Conclusion can be changed to "Several socio-demographic, lifestyle, health-related, and psychological factors were found to be significantly positively and negatively associated with hypertension."

**Reply 5**

**In addition to the above suggestions, the English language editor has suggested some further changes to the abstract. We have revised the abstract to reflect the suggested changes. Also, we have made changes to the conclusion as suggested above.**
